# *VdNUC-2*, the Key Regulator of Phosphate Responsive Signaling Pathway, Is Required for *Verticillium dahliae* Infection

**DOI:** 10.1371/journal.pone.0145190

**Published:** 2015-12-15

**Authors:** Sheng Deng, Cai-yue Wang, Xin Zhang, Qing Wang, Ling Lin

**Affiliations:** Institute of Plant Protection, Jiangsu Academy of Agricultural Sciences, Nanjing, China; University of Nebraska-Lincoln, UNITED STATES

## Abstract

In fungal cells, a phosphate (Pi) responsive signaling and metabolism (PHO) pathway regulates Pi-homeostasis. NUC-2/PHO81 and its homologs are one of the most important components in the regulation pathway. In soil-borne phytopathogenic fungus *Verticillium dahliae*, we identified a *Neurospora crassa nuc-2* homolog gene *VdNUC-2*. VdNUC-2 is composed of 1,018 amino acids, and is highly conserved in tested filamentous fungi. Under conditions of Pi-starvation, compared with the wild-type strain and ectopic complementation strains, the *VdNUC-2* knocked out mutants exhibited reduced radial growth, decreased production of conidia and microsclerotia, and were more sensitive to hydrogen peroxide stress. The virulence of *VdNUC-2* defective mutants was significantly compromised, and that was unable to be restored by exogenous application of extra Pi. Additionally, the deletion mutants of *VdNUC-1*, a key transcription factor gene positively controlled by *VdNUC-2* in the PHO pathway, showed the similar cultural phenotypes as *VdNUC-2* mutants when both of them grew in Pi-limited conditions. However, the virulence of *VdNUC-1* mutants was comparable to the wild-type strain. These evidences indicated that the virulence reduction in *VdNUC-2* mutants is not due to the interruptions in the PHO pathway or the disturbance of Pi-homeostasis in *V*. *dahliae* cytoplasm. *VdNUC-2* is not only a crucial gene in the PHO pathway in *V*. *dahliae*, but also is required for the full virulence during host-infection.

## Introduction

Vascular wilt caused by soil-borne Verticillium dahliae is a destructive disease in a wide range of economically important crops, including cotton, potato, lettuce, tomato, eggplant and strawberry, resulting in huge worldwide crops losses every year [1–2]. In soil, the *V*. *dahliae* microsclerotia, a kind of mycelial resting structure, are the major source of primary infection [2]. When the microsclerotia sense the release of root exudates in the host rhizosphere, the resting structures germinate. The germ tubes and growing mycelia contact the host roots and then combat the host root cells with their special armament, such as cell wall-degrading enzymes and virulence factors [1–3]. After entering the vascular tissues, the fungus produces conidia, which can spread through the sap stream in vessels and result in systemic infection [2].

Recently, great advances have been achieved to increase the knowledge in molecular mechanisms underlying the pathogenicity of *V*. *dahliae*. Klosterman et al. described the first publicly released *Verticillium* genome and identified a glucan glucosyltransferase gene required for full virulence in *Nicotiana benthamiana* [3]. de Jonge et al. identified the first effector gene *Ave1* that activates the resistance response in tomato containing the *Ve1* gene and enhances virulence on susceptible tomato (without *Ve1*) [4]. Subsequently, several other effectors were discovered. These effectors were found to be required for the full virulence of *V*. *dahliae* during infection of tomato and cotton [5,6]. Furthermore, increasing numbers of pathogenicity- or virulence-related genes have been identified, such as *VdRac1*, *Vta2*, *VdCPC1*, *VdSge1*, and *VdSSP1* [7–11]. Notably, except for the effector genes, many scholars found that that the pathogenicity- or virulence-related genes are correlated with vegetative fungal growth [12].

Inorganic phosphate (Pi), an essential nutrient in living cells, participates in numerous important cellular biological processes, such as nucleic acid synthesis, construction of the cellular membrane system, energy transport, and cellular signal transduction. Thus, maintaining Pi homeostasis in cells is crucial for microbial survival. In the filamentous fungus *Neurospora crassa*, a phosphate-responsive signaling and metabolic pathway known as the PHO pathway has been identified [13,14] and excellently reviewed by Tomar and Sinha [15]. The pathway involves a core regulation system composed of at least four genes, namely, *nuc-2*, *preg*, *pgov*, and *nuc-1*. In the genetic hierarchy, *nuc-2* is the most upstream gene regulated in response to extracellular Pi changes [13,14,16]. Under Pi shortage, NUC-2 perceives low Pi levels in some unknown ways and transmits the signals downstream by inhibiting the PREG–PGOV complex function [14–16]. This occurrence allows the translocation of the key transcription factor NUC-1 into the nucleus and the activation of the expression of the Pi-responsive genes [14,15].

Many important genes in conserved signaling pathways or nutrient metabolic processes are essential for the full virulence of phytopathogenic fungi. These pathways include the protein kinase pathways [17,18], G protein and small GTPase protein family pathways [7,19–21], carbon and nitrogen metabolism [11,22,23], and amino acid and vitamin syntheses [9,24]. However, the key gene involved in the PHO pathway that plays an important role in the pathogenicity of filamentous fungi is rarely studied. In the present work, we identified a *nuc-2* homolog gene in *V*. *dahliae*, named as *VdNUC-2*, which not only controls Pi homeostasis under low Pi conditions but also regulates the key steps of host infection.

## Results

### 1. Identification of the *VdNUC-2* gene

**Fig 1:**
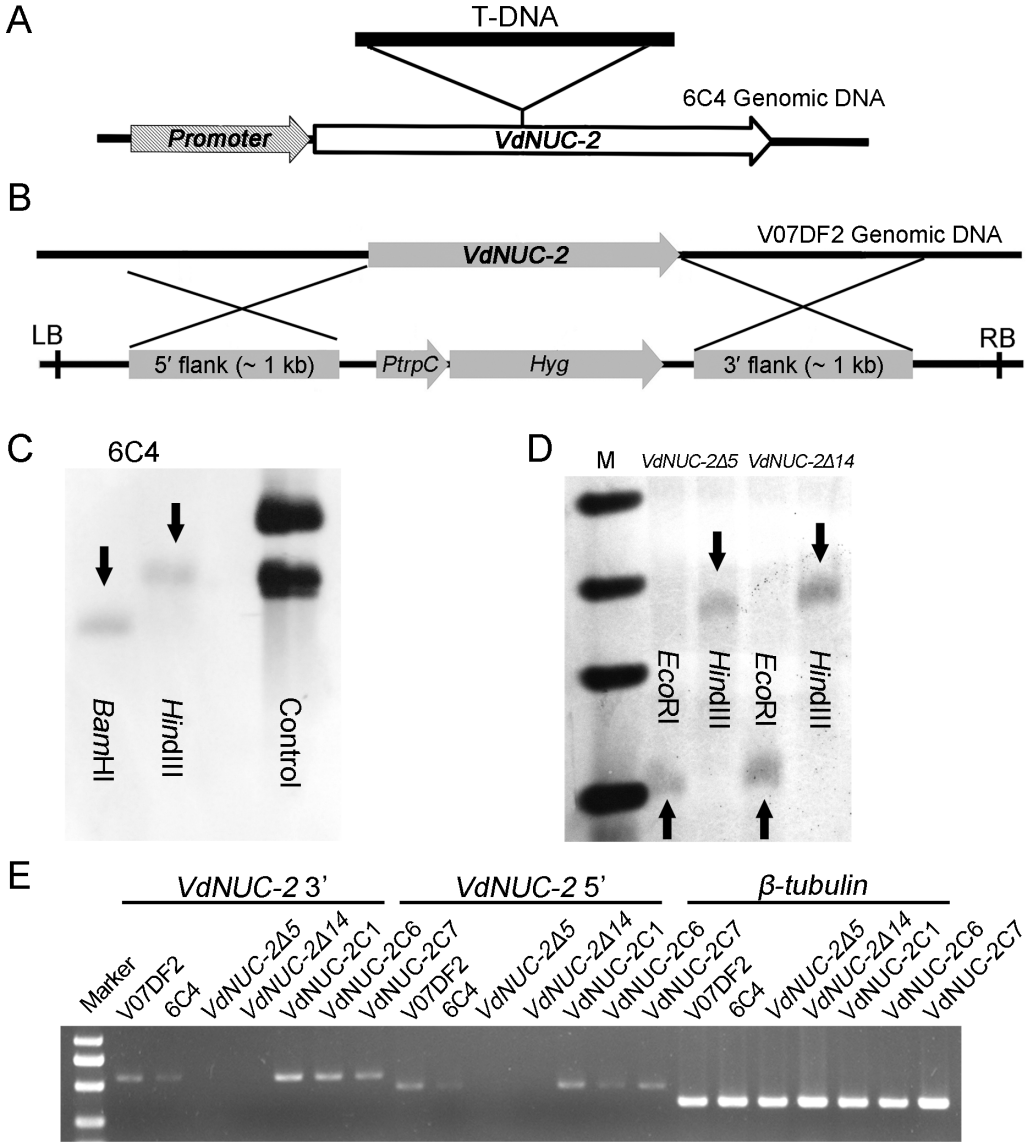
Identification of the *VdNUC-2* gene in mutants and complementation strains. A, In mutant 6C4, the exon of *VdNUC-2* is truncated by a T-DNA insertion. C, T-DNA insertional copy number was confirmed by Southern blot in 6C4. Genomic DNA was digested by *Hin*dIII and *Bam*HI. B, Schematic of targeted deletion in mutants *VdNUC-2Δ5* and *VdNUC-2Δ14*. D, Copy number of T-DNA insertion was also confirmed by Southern blot in the two deletion mutants. Their genomic DNA was digested by *Eco*RI and *Hin*dIII. E, The expression levels of *VdNUC-2* in all test strains were analyzed by semi-quantitative PCR. *VdNUC-2* 3′ and *VdNUC-2* 5′ indicate the primer pairs bound at the 3′ and 5′ ends, respectively, of *VdNUC-2* cDNA. *β-tubulin* was used as an internal reference gene. V07DF2 indicates the wild-type strain; VdNUC-2C1, VdNUC-2C6, and VdNUC-2C7 denote the three ectopic complementation strains.

In our T-DNA random insertional mutagenesis library, a mutant named 6C4 was found due to the reduction of virulence in cotton seedlings [25]. The results obtained from Southern blot, thermal asymmetric interlaced polymerase chain reaction (TAIL-PCR), and BLAST search indicated that a single copy of T-DNA was inserted in the exon region of a putative gene, which was tentatively called *V*. *dahliae NUC-2* (*VdNUC-2*) (Fig 1A and 1C and S1 Fig). After BLAST search against the genome data, only one homolog gene, particularly VDAG_00896.1 (encoding ankyrin repeat protein nuc-2), was found in the genome of *V*. *dahliae* strain VdLs17.

For further investigation of the function of *VdNUC-2*, targeted deletion mutants and complementation strains were generated based on the *Agrobacterium tumefaciens*-mediated transformation (ATMT) method. The results of Southern blot and semi-quantitative PCR confirmed that the *VdNUC-2* gene was knocked out by a single copy of T-DNA integrated in both targeted deletion mutants *VdNUC-2Δ5* and *VdNUC-2Δ14* (Fig 1B, 1D and 1E). Additionally, the results of semi-quantitative PCR indicated that the expression of the *VdNUC-2* gene was restored in the ectopic transformants VdNUC-2C1, VdNUC-2C6, and VdNUC-2C7 (Fig 1E). Two of these transformants were adopted for further analysis in this study.

**Fig 2:**
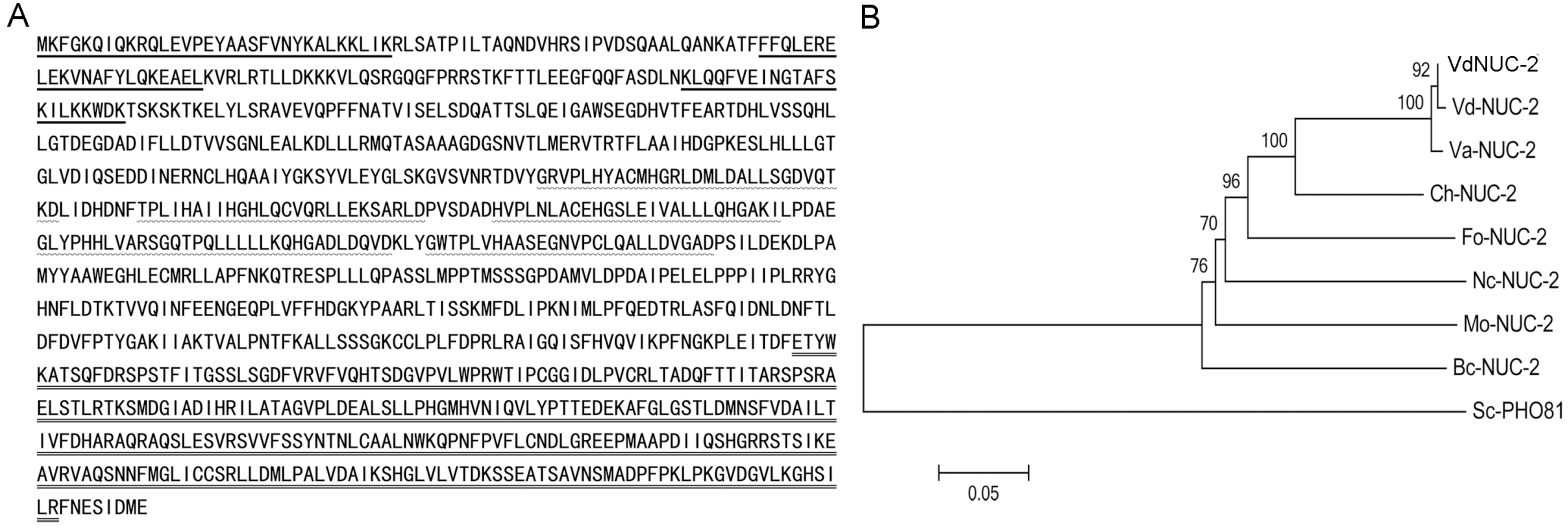
Deduced amino acid sequence of VdNUC-2 and phylogenetic tree of VdNUC-2 with homologs from other fungal species. A, Deduced amino acid sequence of VdNUC-2. The underlined N-terminus sequences indicate the SPX domain. The middle sequences marked by wavy lines represent the five putative ankyrin repeats. The C-terminus indicated by a dotted line shows the catalytic domain of the PI-PLCc_GDPD_SF superfamily. B, Phylogenetic relationships of VdNUC-2 with homologs from other fungal species. VdNUC-2: *V*. *dahliae* V07DF2; Vd-NUC-2: *V*. *dahliae* VdLs.17 (VDAG_00896T0); Va-NUC-2: *V*. *alfalfae* VaMs.102 (VDBG_03666T0); Ch-NUC-2: *Colletotrichum higginsianum* (CCF40876); Fo-NUC-2: *Fusarium oxysporum* f. sp. Melonis 26406 (EXK46788); Nc-NUC-2: *Neurospora crassa* (AAB03277); Mo-NUC-2: *Magnaporthe oryzae* 70–15 (XP_003709322); Bc-NUC-2: *Botrytis cinerea* BcDW1 (EMR88825); Sc-PHO81: *Saccharomyces cerevisiae* S288c (NP_011749).

Based on the cDNA sequence obtained by RACE and on BLAST results retrieved from the National Center for Biotechnology Information (NCBI) databases, we found that the *VdNUC-2* gene possessed an open reading frame of 3057 nucleotides (GenBank database accession no. KT454782) and encoded a protein of 1018 amino acids. In the putative sequence of VdNUC-2, three conserved domains were present: the SPX domain (pfam03105), ankyrin repeat domain (cd00204), and catalytic domain of the PI-PLCc_GDPD_SF superfamily (cl14615) (Fig 2A). Eight orthologs from other fungi obtained from the NCBI database were compared with the deduced amino acid sequence of VdNUC-2. Their alignments are shown in S2 Fig. Moreover, a phylogenetic relationship tree was drawn for the analyzed orthologs (Fig 2B). Except Sc-PHO81 in *Saccharomyces cerevisiae* S288c, all the other orthologs were highly conserved in both plant pathogenic and nonpathogenic fungi, including *Colletotrichum higginsianum*, *Fusarium oxysporum*, *Magnaporthe oryzae*, *Botrytis cinerea*, and *N*. *crassa*.

### 2. *VdNUC-2* is required for radial growth and conidia production in Pi-deficient culture conditions

**Fig 3:**
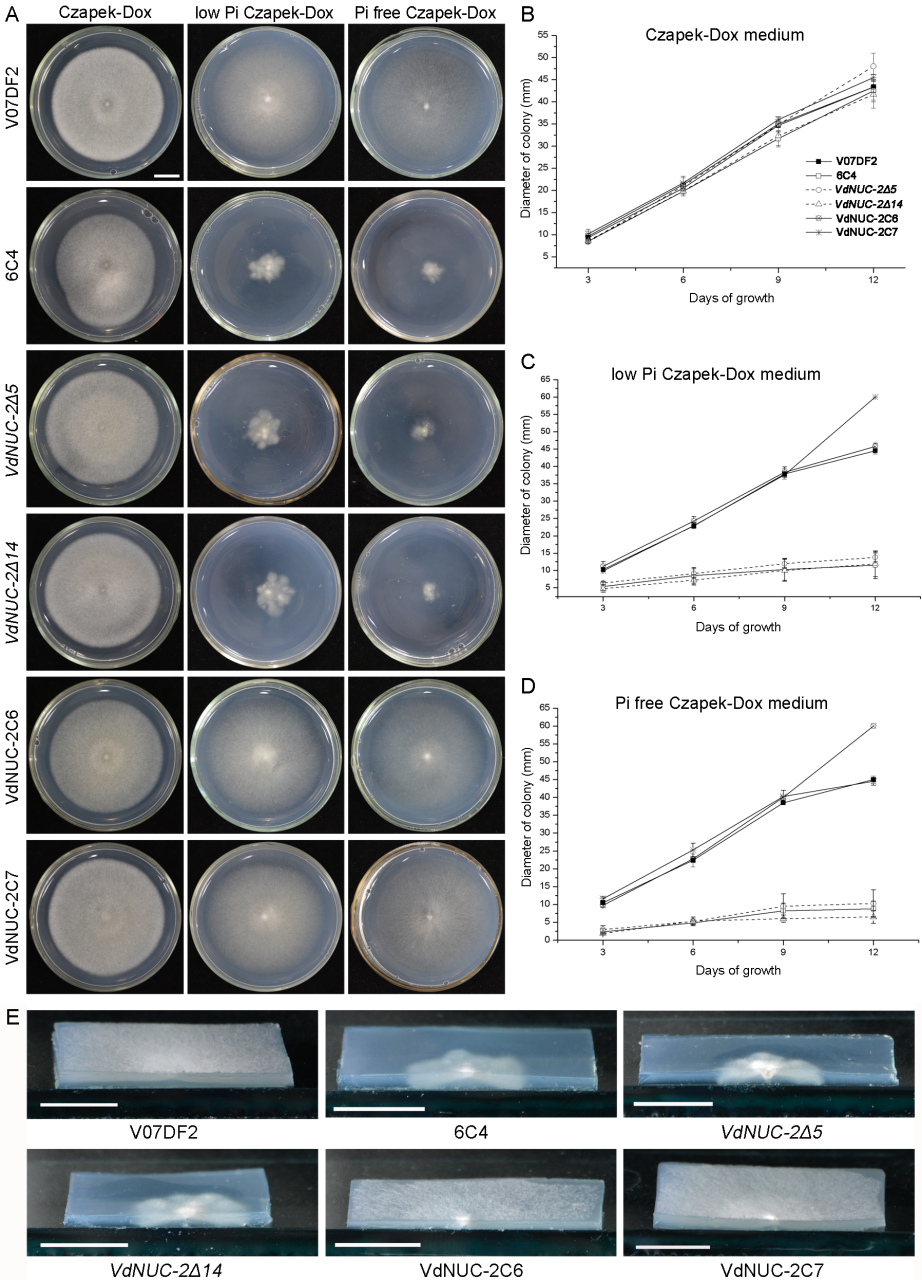
Mutation of *VdNUC-2* resulting in altered colony morphology in Pi-deficient Czapek–Dox media. A, Colony morphologies of wild-type *V*. *dahliae* (V07DF2), T-DNA insertion mutant (6C4), two targeted deletion mutants (*VdNUC-2Δ5* and *VdNUC-2Δ14*), and two complementation strains (VdNUC-2C6 and VdNUC-2C7) after 14 days of incubation on normal Pi (Con _Pi_ = 5.7 mM), low Pi (Con _Pi_ = 57 μM), and Pi-free Czapek–Dox (Con _Pi_ = 0 mM) media. B, C, and D, Colony diameters measured at indicated days after inoculation on culture media containing different Pi contents. Error bars represent standard deviation (*n* = 4). E, Reduced aerial hyphae growth in *VdNUC-2* mutants under low Pi culture conditions. The pictures were obtained after 14 days of incubation. Bar = 1 cm.

**Fig 4:**
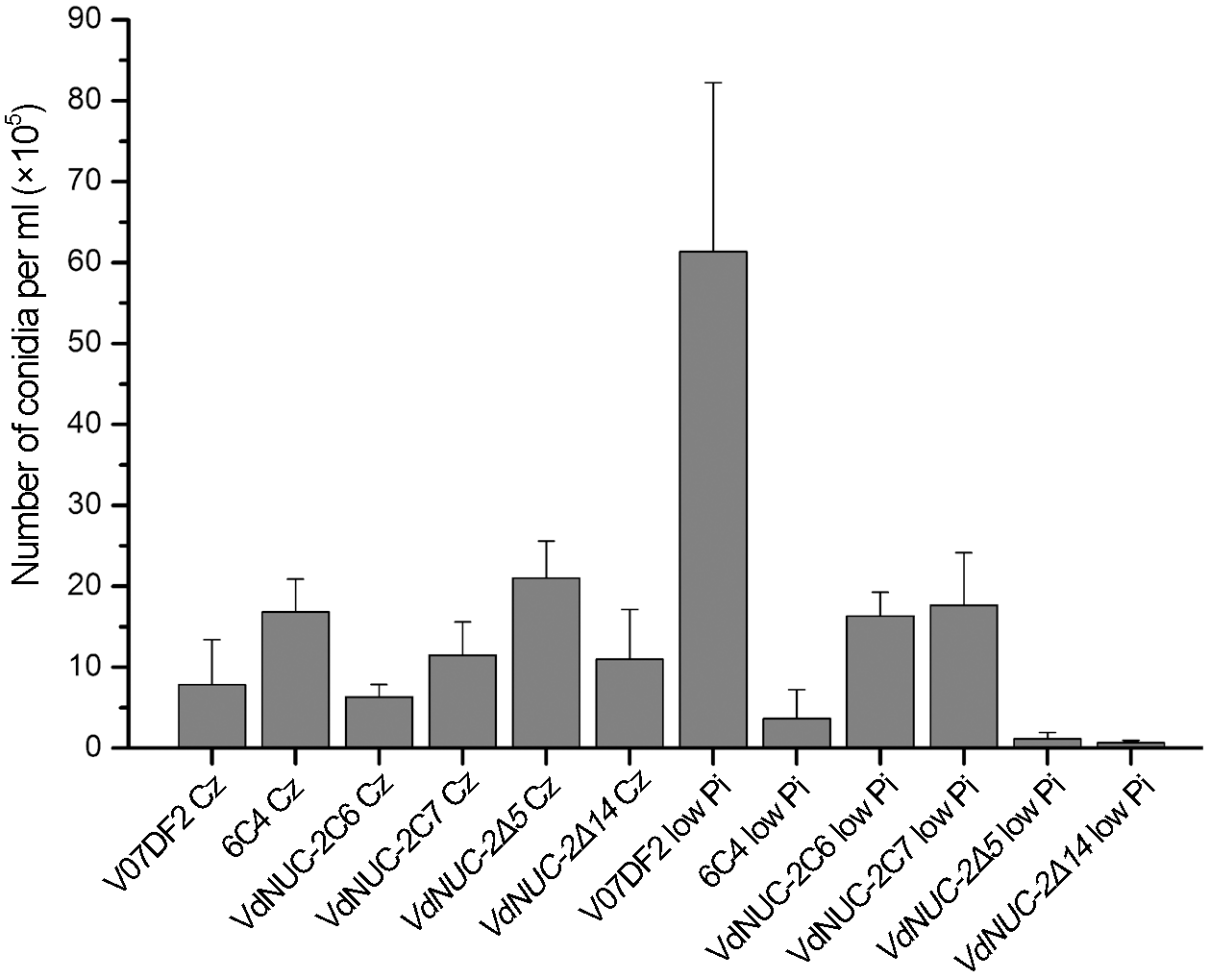
Reduced conidia production in *VdNUC-2* mutants cultured in low Pi Czapek–Dox liquid media. Cz and low Pi indicate the normal (Con _Pi_ = 5.7 mM) and low Pi Czapek–Dox liquid media (Con _Pi_ = 57 μM), respectively. The data were collected two weeks after incubation. Error bars represent the standard deviation of three biological replicates.

The *NUC-2* homolog in *N*. *crassa* has been shown to be involved in sensing phosphate availability and transmitting signals downstream to the regulatory pathway [13]. In the present study, the gene functions in *V*. *dahliae* were investigated using three *VdNUC-2* mutants, two corresponding ectopic complementation transformants, and the wild-type strain V07DF2. These samples were incubated on Czapek–Dox medium plates and Pi-deficient Czapek–Dox medium plates (Fig 3A). The fungi grew normally on the Czapek–Dox plates (Pi concentration, Con _Pi_ = 5.7 mM). By contrast, on the low Pi culture media (Con _Pi_ = 57 μM), 6C4 and two other targeted deletion mutants showed significantly reduced radial growth compared with the wild-type strain and ectopic transformants. On the Pi-free Czapek–Dox plates, the three *VdNUC-2* mutants were difficult or even impossible to grow, whereas the radial growth of the wild-type strain and ectopic transformants were not obviously affected (Fig 3A–3D). In the V07DF2 and ectopic transformants, the low Pi levels only generated thin aerial hyphae on the fungal colonies but did not lead to radial growth reduction (Fig 3A–3D). Interestingly, in the low Pi Czapek–Dox media, the *VdNUC-2* mutants showed more vigorous substrate mycelia compared with V07DF2 and the ectopic transformants (Fig 3E). A compensatory mechanism was probably activated to help fungi acquire as much phosphate as possible when their cells were suffering Pi starvation stress. In addition, when cultured under low Pi conditions, conidia production of the *VdNUC-2* mutants was significantly diminished because of Pi starvation (Fig 4). Thus, *VdNUC-2* participated in colony morphogenesis and conidia production when *V*. *dahliae* grew in Pi-deficient conditions.

### 3. Expression of downstream phosphate transporter genes in the *VdNUC-2* mutants is significantly compromised in Pi-deficient culture conditions

In *S*. *cerevisiae*, five Pi transporters are encoded, namely, Pho84, Pho89, Pho87, Pho90, and Pho91 [26]. Among these transporters, Pho84 and Pho89 are high-affinity Pi uptake transporters activated in Pi-limited conditions. Meanwhile, Pho87, Pho90, and Pho91 comprise the low-affinity Pi uptake systems that play major roles in the phosphate homeostasis of yeast under high Pi conditions [26]. The open reading frame sequences of *PHO84* (D90346), *PHO87* (NM_001178751), *PHO89* (NM_001178644), and *PHO90* (NM_001181631) were used as query sequences for BLASTx search in the *Verticillium* group protein database at the Broad Institute website (http://www.broadinstitute.org/annotation/genome/verticillium_dahliae/MultiHome.html). In *V*. *dahliae* VdLs.17, six homolog genes were found, namely, a *PHO84* homolog (VDAG_03222.1), a *PHO87* and *PHO90* homolog (VDAG_03960.1), and four *PHO89* homologs (VDAG_07630.1, VDAG_03800.1, VDAG_04027.1, and VDAG_07583.1).

**Fig 5:**
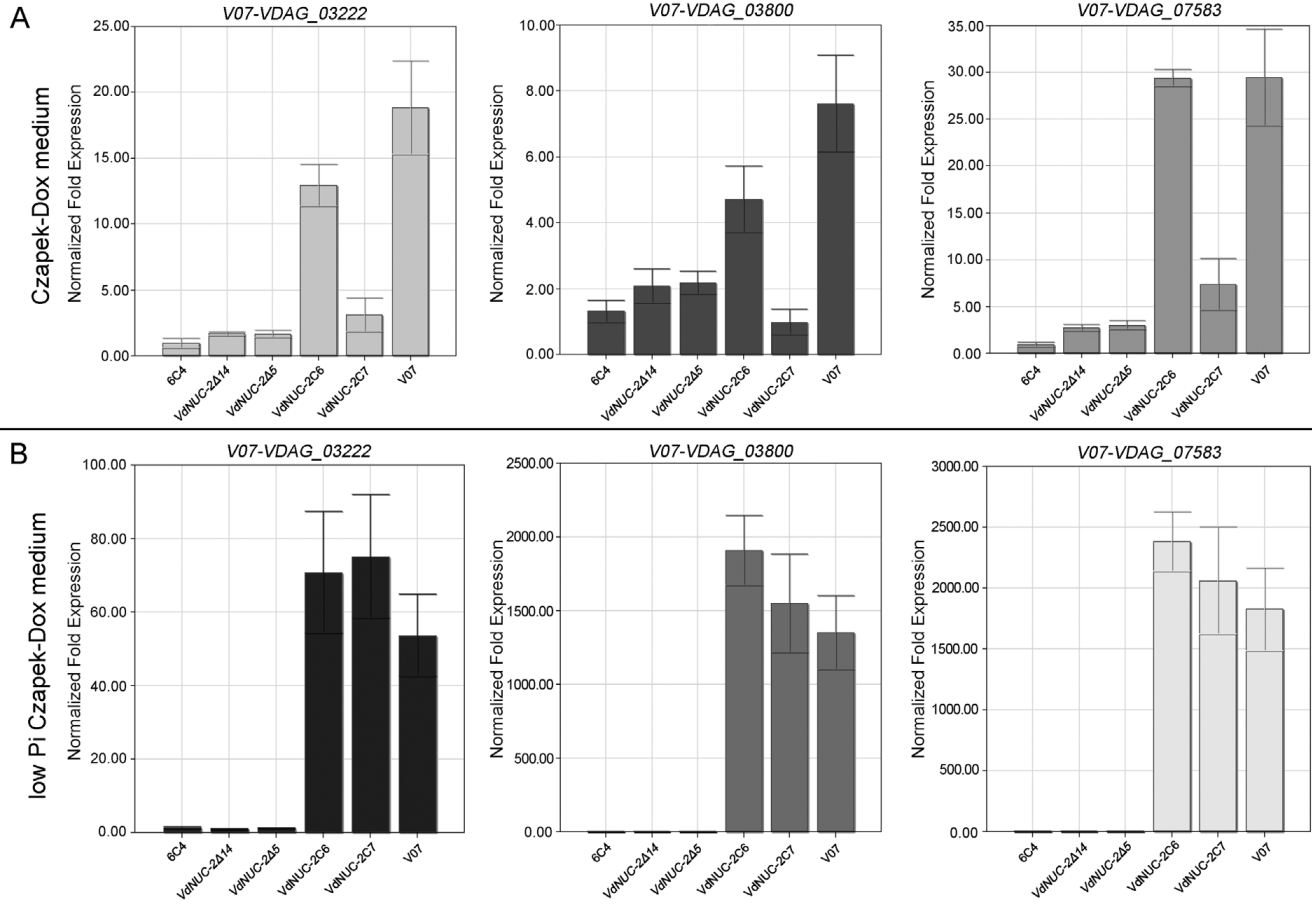
Expression levels of the putative Pi transporter genes in the *VdNUC-2* mutants and complementation strains. Quantitative real-time PCR was employed to measure gene expression levels in the wild-type strain (V07), T-DNA insertion mutant (6C4), two targeted deletion mutants (*VdNUC-2Δ5* and *VdNUC-2Δ14*), and two ectopic complementation strains (VdNUC-2C6 and VdNUC-2C7). A, Expression of three putative Pi transporter genes in the tested strains cultured on normal Czapek–Dox plates (Con _Pi_ = 5.7 mM). *V07-VDAG_03222*: inorganic phosphate transporter gene in V07DF2 corresponding to VdLs.17 homolog VDAG_03222; *V07-VDAG_07583*: phosphate-repressible phosphate permease gene in V07DF2 corresponding to VdLs.17 homolog VDAG_07583; *V07-VDAG_03800*: phosphate-repressible phosphate permease gene in V07DF2 corresponding to VdLs.17 homolog VDAG_03800. B, Expression levels of the three genes in the tested strains cultured on low Pi Czapek–Dox plates (Con _Pi_ = 57 μM).

The expression of these homolog genes was analyzed in our tested strain that grew in normal or low Pi conditions. VDAG_03960.1, VDAG_07630.1, and VDAG_04027.1 were probably the low-affinity Pi uptake transporters, because these homologs exhibited similar expression levels under all circumstances in our tested strains (data not shown). By contrast, VDAG_03222.1, VDAG_03800.1, and VDAG_07583.1 likely comprised the high-affinity Pi uptake transporters because their expression levels were significantly affected by *VdNUC-2* deletion under low Pi conditions (Fig 5). Additionally, the expression levels of *VdNUC-2* and its four putative downstream genes *VdNUC-1* (VdLs.17 homolog VDAG_03154), *VdPHO-2* (VdLs.17 homolog VDAG_01304), *VdPHO-3* (VdLs.17 homolog VDAG_06414), and *VdPREG* (VdLs.17 homolog VDAG_06766) were also analyzed under the same conditions (S3 Fig). Compared with the wild-type strain and two ectopic transformants, the expression levels of *VdNUC-2*, *VdPHO-2*, and *VdPRE*G in the *VdNUC-2* knock-out mutants were suppressed under low Pi conditions (S3 Fig). However, all strains showed similar expression levels of *VdNUC-1* and *VdPHO-3* under the same conditions (S3 Fig). The expression of the two genes was likely controlled by different regulatory pathways. These results indicated that *VdNUC-2* was a key regulator of the phosphate-responsive signaling pathway in *V*. *dahliae*.

### 4. *VdNUC-2* is required for *V*. *dahliae* infection

**Fig 6:**
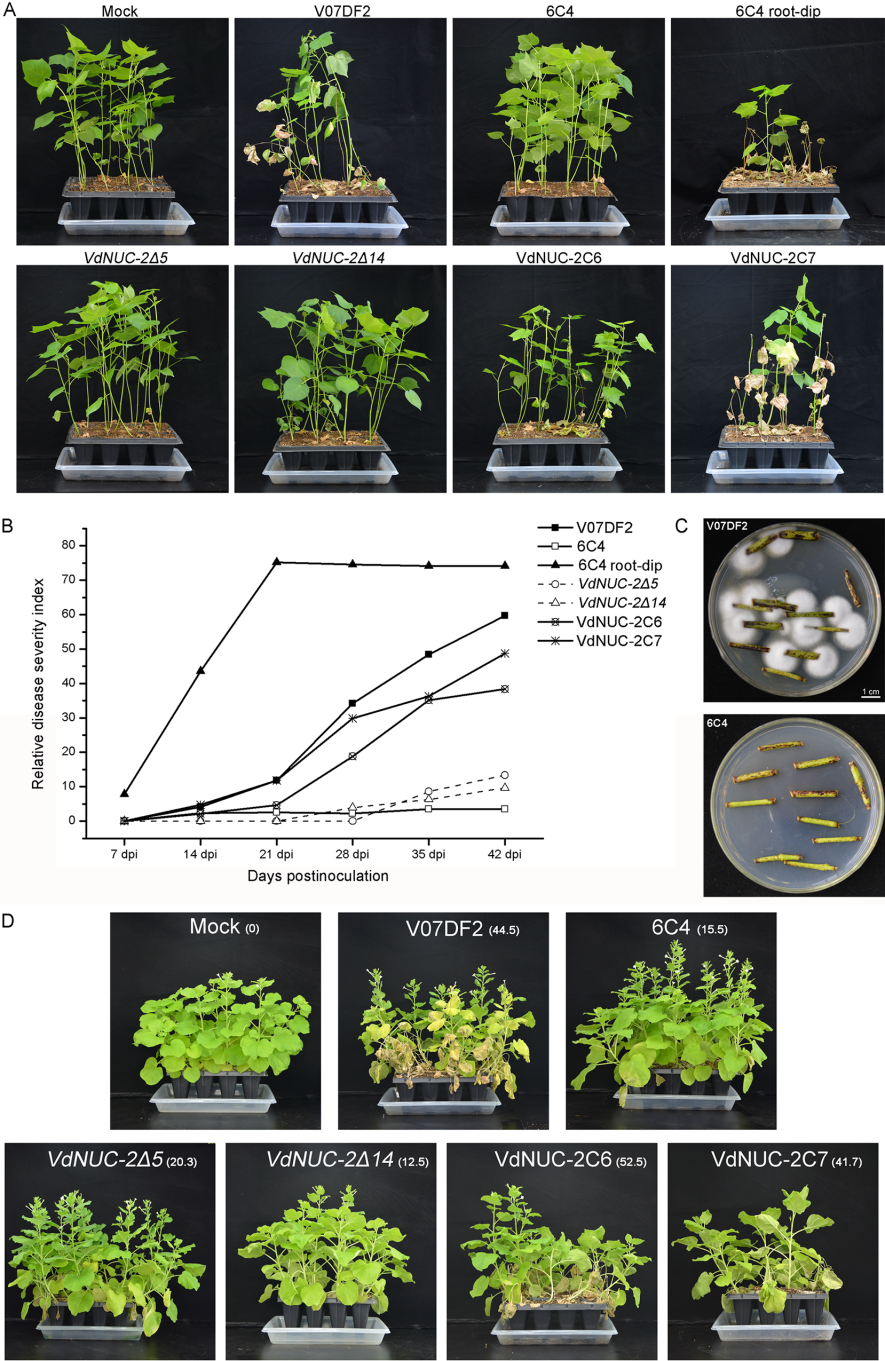
Virulence and infection assessment of the *VdNUC-2* mutants and complementation strains. A, Cotton seedlings infection assays. The mock (water treatment) and V07DF2 (wild-type strain) were considered as negative and positive controls, respectively. The 6C4 root dip indicated that the seedlings were uprooted carefully and then dipped in a conidial suspension of 6C4 (1 × 10^7^ conidia/ml). The seedlings were then replanted in fresh culture soil. Other inoculations (6C4, *VdNUC-2Δ5*, *VdNUC-2Δ14*, VdNUC-2C6, and VdNUC-2C7) were performed by root irrigation around the plant stem base using the conidial suspension of each strain (1 × 10^7^ conidia/ml). B, Assessment of disease development by relative disease severity index as described by Deng et al. [25] (*n* ≥ 36). C, Fungal regrowth examination. Image of fungal hyphae growing on cotton seedlings stem pieces infected by the wild-type strain and *VdNUC-2* mutant 6C4. D, Tobacco (*Nicotiana benthamiana*) seedlings infection assays. All treatments were performed by root irrigation with conidial suspension (1 × 10^7^ conidia/ml). The relative disease severity indices are presented next to each treatment name with subscript characters.

Previous research on *NUC-2* and its homologs focused on the PHO pathway in *N*. *crassa* and *S*. *cerevisiae* [14,15]. However, in plant pathogenic fungi, little knowledge was revealed on the functions of the homolog genes. In the present study, we assessed the role of *VdNUC-2* in *V*. *dahliae* pathogenicity in cotton and tobacco seedlings. After employing the root irrigation inoculation method, the T-DNA insertion mutant 6C4 and two targeted deletion mutants hardly caused disease in cotton and tobacco seedlings. By contrast, the ectopic transformants and the wild-type strain caused severe *Verticillium* wilt symptoms in the seedlings (Fig 6). However, when 6C4 was inoculated on wounded root cotton seedlings through the root-dip method, the disease symptoms developed more quickly than that caused by the wild-type strain inoculation (Fig 6A and 6B). These results indicated that the *VdNUC-2* mutation resulted in failure to penetrate host physical barriers, which reduced the virulence of the pathogen.

### 5. Exogenous application of extra Pi failed to restore the virulence of *VdNUC-2* mutant 6C4 in cotton seedlings planted in nutrient solution

**Fig 7:**
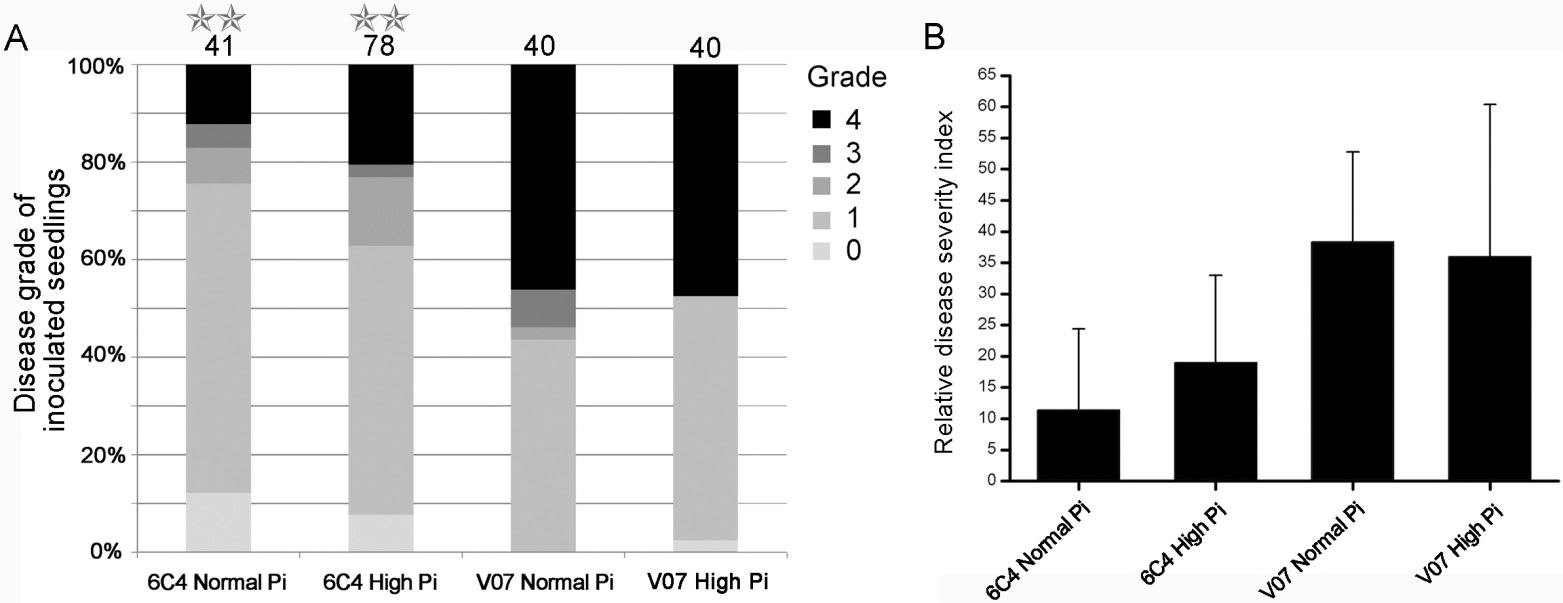
Pathogenicity assays on cotton seedlings planted in nutrient solution. Normal Pi refers to the ordinary Hoagland hydroponic nutrient solution (Con _Pi_ = 1 mM), whereas high Pi indicates the modified Hoagland nutrient solution with high Pi content (Con _Pi_ = 6 mM). The seedlings were separately treated by V07 (wild-type strain V07DF2) and 6C4 (T-DNA insertion mutant). A, Disease grades of inoculated plants under different treatments (percentage stacked column chart, *P* < 0.01, Fisher’s exact test). B, The relative disease severity index corresponds to each treatment in A. The values for the relative disease severity indices were calculated for each treatment, and the error bars indicate the standard deviations calculated from 6 to 12 replicates. Three to eight seedlings were included in each replicate.

We have demonstrated that *VdNUC-2* is a key regulator of PHO pathway as well as its *N*. *crassa* homolog *NUC-2*. We also showed that *VdNUC-2* is indispensable in *V*. *dahliae* infection. Thus, we aimed to decipher whether any connections exist between Pi homeostasis and the virulence of *V*. *dahliae*. To modulate the available Pi levels in vitro after inoculation, we applied the Hoagland hydroponic nutrient solution for the soilless culture of cotton seedlings. When the second true leaves had expanded, the cotton seedlings were gently transferred into the nutrient solution with normal (1 mM) or high (6 mM) Pi contents containing 1 × 10^7^ conidia/mL for inoculation. Seven to eight weeks later, the disease symptoms were examined and the disease severity index was obtained (Fig 7 and S7 Fig). For the mutant 6C4, no significant difference in disease grade was found between the normal and high Pi treatment groups. Compared with the wild-type strain treatments, the relative disease severity index of both 6C4 treatments was much lower. The results suggested that the virulence reduction of *VdNUC-2* mutant 6C4 was caused by some other reason but not the Pi levels in vitro or in vivo.

### 6. *VdNUC-1* and its controlled PHO pathway are not required for *V*. *dahliae* infection

**Fig 8:**
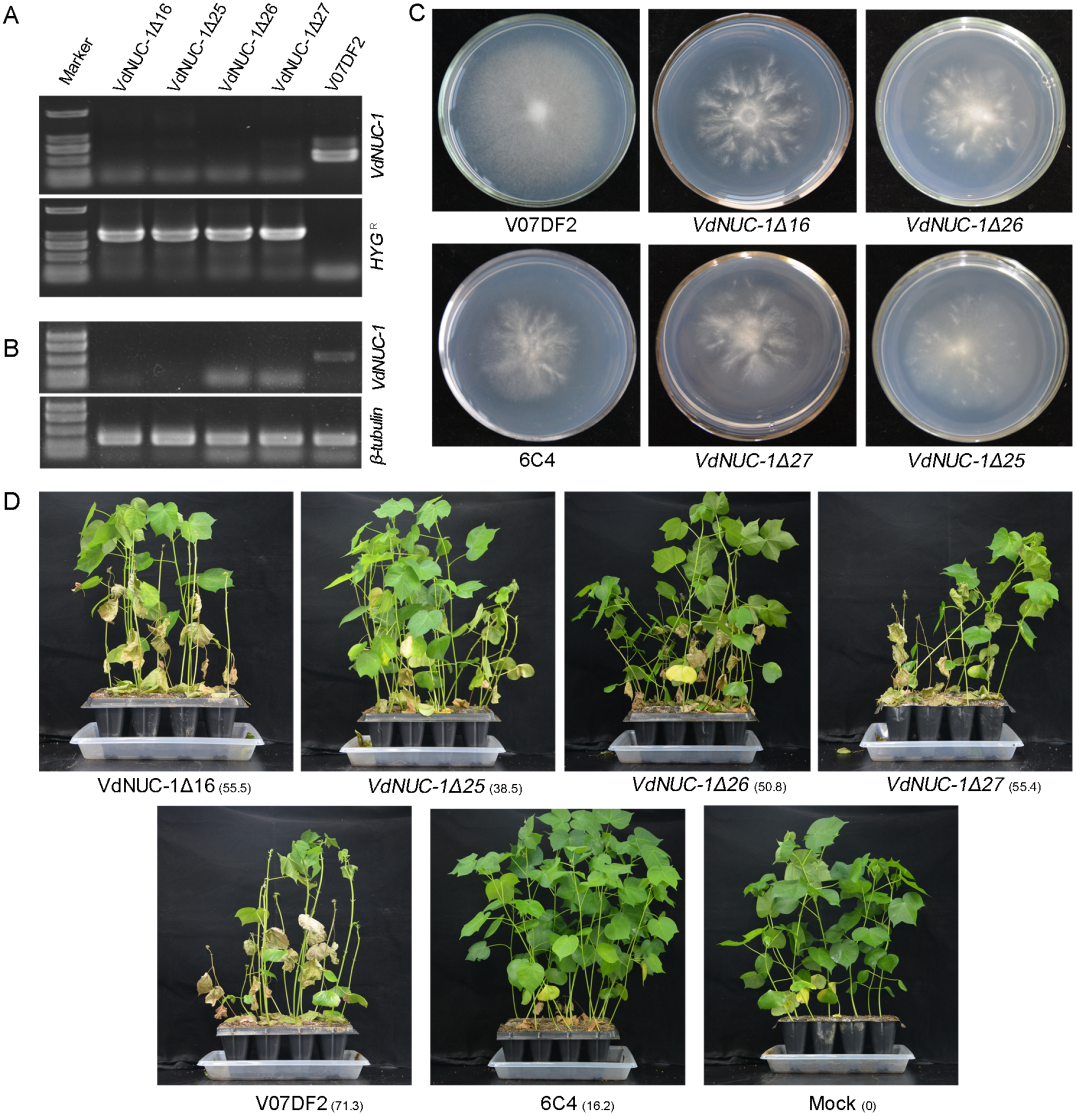
Identification of *VdNUC-1* targeted deletion mutants. A, Detection of the *VdNUC-1* gene in the genome of four deletion mutants by PCR. Top panel: analysis of targeted gene *VdNUC-1*; bottom panel: detection of hygromycin B resistance gene (*HYG* ^*R*^). *VdNUC-1Δ16*, *Δ25*, *Δ26*, *Δ27*, and V07DF2 indicate the four deletion mutants and the wild-type strain, respectively. B, Expression levels of *VdNUC-1* in all the test strains as analyzed by semi-quantitative PCR. C, Wild-type strain V07DF2, *VdNUC-2* mutant 6C4, and the four *VdNUC-1* deletion mutants grown on low Pi Czapek–Dox solid media (Con _Pi_ = 57 μM). D, Virulence assay of *VdNUC-1* deletion mutants. The wild-type strain and *VdNUC-2* mutant 6C4 were set as the controls. Three biological repetitions were conducted, and at least 13 seedlings were treated in each repetition. Pictures were obtained seven weeks after inoculation. The relative disease severity index is presented next to each treatment name with subscript characters.

*N*. *crassa* NUC-1 and *S*. *cerevisiae* PHO-4 are the key transcription regulators in the PHO pathway; they control the expression of numerous Pi starvation-responsive genes [14,15,27,28]. The amino sequence of *N*. *crassa* NUC-1 (AAA33603.1) was used for BLASTx search in the *Verticillium* group protein database. Only one homolog protein VDAG_03154T0, tentatively named as VdNUC-1, was found with 38% identity. The expression of *VdNUC-1* was not affected by *VdNUC-2* mutation during growth at low or normal Pi concentrations (S3 Fig). Four *VdNUC-1* deletion mutants were obtained (Fig 8A and 8B). These mutants, as well as the *VdNUC-2* mutant 6C4, exhibited reduced radial growth during incubation in low Pi Czapek–Dox media (Fig 8C). These results suggested that both *VdNUC-2* and its putative downstream gene *VdNUC-1* were key regulators of the PHO pathway in *V*. *dahliae*. Subsequently, the virulence of the *VdNUC-1* deletion mutants toward cotton seedlings was assessed by the root irrigation inoculation method. Seven weeks after inoculation, severe disease symptoms were observed on the cotton seedlings inoculated with the wild-type strain and four *VdNUC-1* deletion mutants but not on those inoculated with the *VdNUC-2* mutant 6C4 (Fig 8D). The above results demonstrated that the virulence of *V*. *dahliae* was neither dependent on the PHO pathway nor Pi homeostasis controlled by *VdNUC-1*.

### 7. Inorganic phosphate is indispensable for microsclerotial development

**Fig 9:**
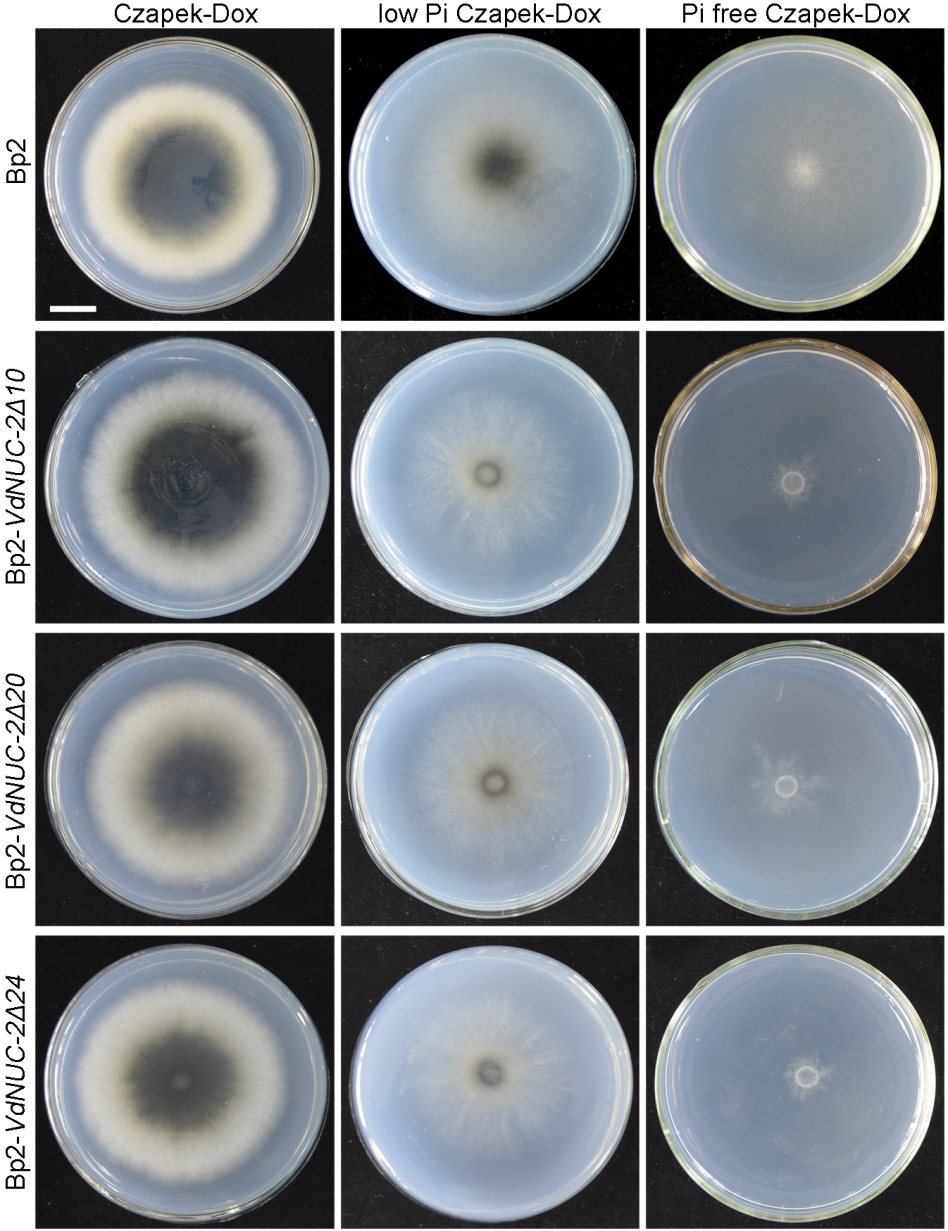
Severe inhibition of microsclerotia formation by *VdNUC-2* mutation in *V*. *dahliae* under Pi-deficient culture conditions. Bp2, the other wild-type strain of *V*. *dahliae*; Bp2-*VdNUC*-*2Δ10*, *Δ20*, and *Δ24* refer to the three *VdNUC-2* targeted deletion mutants in Bp2. All of these mutants were cultured on normal (Con _Pi_ = 5.7 mM) and Pi-deficient Czapek–Dox media plates (Con _Pi_ = 57 μM). To investigate strains’ microsclerotial development, 5 μL of spore suspension (1 × 10^7^ spores/mL) of each strain was dripped onto the center of the plates. Pictures were taken after two weeks of incubation. Bar = 1 cm.

To examine the roles of *VdNUC-2* and inorganic phosphate in microsclerotial development, the other wild-type *V*. *dahliae* strain Bp2 was introduced. This strain can form normal melanized microsclerotia in Czapek–Dox agar media. Three targeted deletion mutants with compromised virulence (data not shown) and Bp2 were cultured on normal Czapek–Dox media, low Pi media, and Pi-free media, respectively (Fig 9). Compared with the strains cultured on normal Czapek–Dox media, none of the strains incubated in Pi-free media produced visible black microsclerotia (Fig 9). In low Pi Czapek–Dox media, microsclerotia formation of the deletion mutants was significantly compromised, whereas the wild-type strain Bp2 could still produce a considerable amount of microsclerotia (Fig 9). These results demonstrated that the accumulation of sufficient inorganic phosphate in *V*. *dahliae* cells was crucial for microsclerotia formation.

### 8. VdNUC-2 localizes in the cytoplasm and nucleus

**Fig 10:**
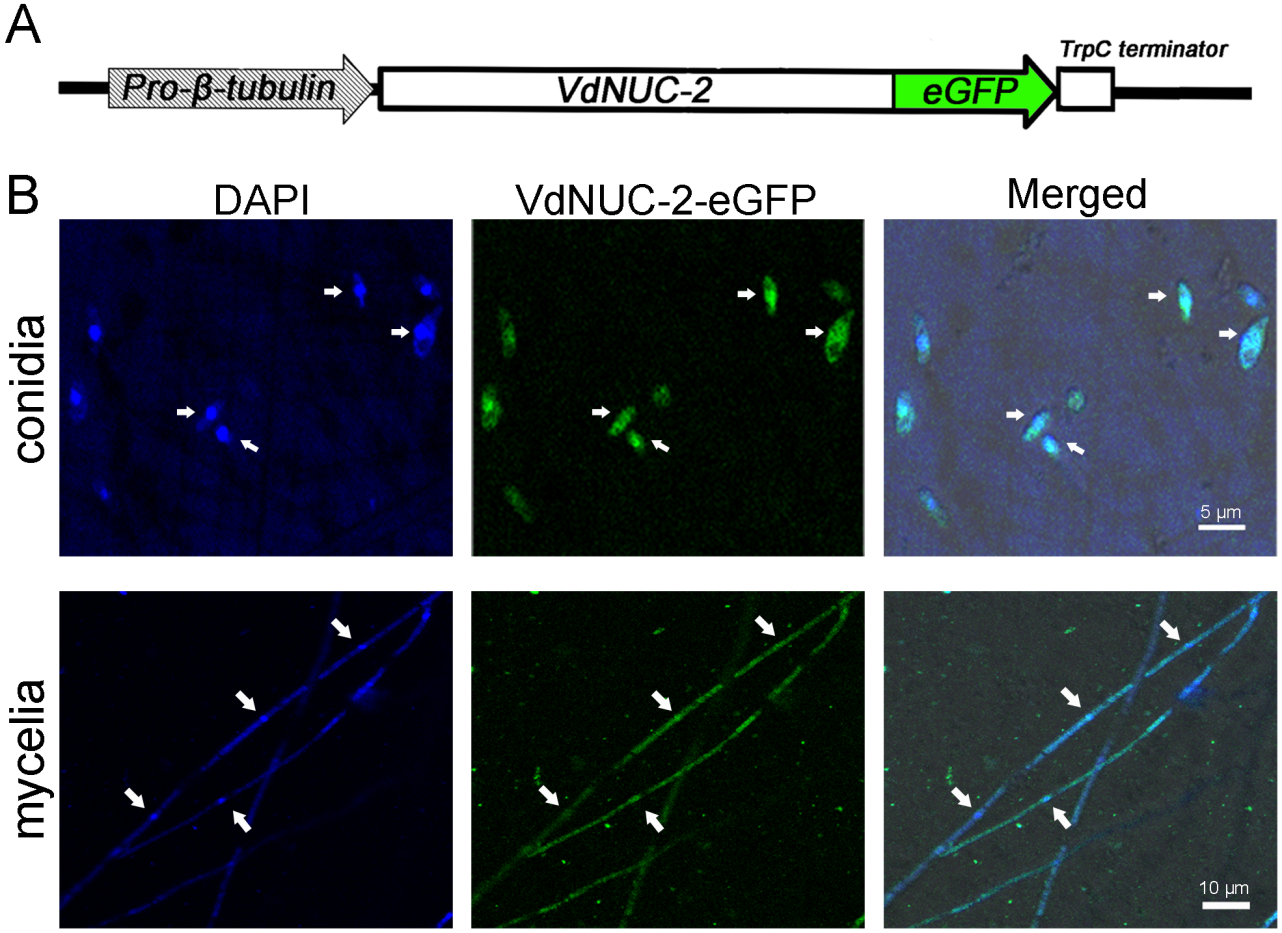
Analysis of cellular localization of the VdNUC-2–eGFP fusion protein. A, Schematic of the overexpression module of VdNUC-2–eGFP in Ti vector. *Pro*-*β-tubulin* indicates the promoter of the *β-tubulin* gene. B, Cellular localization of VdNUC-2–eGFP examined by confocal fluorescent microscopy (Carl Zeiss LSM710). The white arrows indicate the cell nuclei.

To determine the localization of VdNUC-2 in *V*. *dahliae*, an overexpression Ti vector was constructed as shown in Fig 10A. The expression of the *VdNUC-2–eGFP* fusion transcript was controlled by the endogenous constitutive promoter *Pro-β-tubulin*, because the native promoter of *VdNUC-2* failed to accumulate sufficient green fluorescent protein (GFP) fusion protein for observation (data not shown). *Pro-β-tubulin* was derived from the promoter of the *β-tubulin* gene (571 bp upstream region of ATG) in wild-type strain V07DF2. Staining of conidia and mycelia with 4′,6-diamidino-2-phenylindole (DAPI) marked the nucleus of the cells (Fig 10B). Meanwhile, the green fluorescent signal of the VdNUC-2–eGFP fusion protein was found in the nucleus and cytoplasm (Fig 10B). The results indicated that VdNUC-2 localized in both the cytoplasm and nucleus.

## Discussion

Living organisms have evolved sophisticated signal transduction networks that maintain nutrient levels for metabolism and acquisition. The Pi homeostasis regulation system, known as the PHO pathway, has been extensively studied in *S*. *cerevisiae* and *N*. *crassa* [15]. In *S*. *cerevisiae*, PHO81, PHO80, PHO85, and PHO4 showed homology corresponding to the NUC-2, PREG, PGOV, and NUC-1 proteins in *N*. *crassa*. Furthermore, partially similar regulation patterns were found in the two species [15]. To date, besides their roles in the PHO pathway, NUC-2 orthologs serve other functions that are not as frequently addressed, especially in phytopathogenic fungi. In the present study, we identified a *nuc-2* homolog gene, named *VdNUC-2*, in *V*. *dahliae*. The phylogenetic relationship tree and alignment analysis demonstrated that VdNUC-2 was highly conserved compared with the other NUC-2 orthologs (Fig 2B and S2 Fig). Moreover, the transcription level of *VdNUC-2* was induced during growth on low Pi culture media; the expression of *VdNUC-1*, a crucial transcript factor downstream of *VdNUC-2*, was not affected by Pi starvation treatments or *VdNUC-2* deletion (S3 Fig). Similar expression patterns of *nuc-2* and *nuc-1* in *N*. *crassa* were found in previous research [16,28]. These limited results suggested that the NUC-2 orthologs sensed and transduced signals in analogous ways in filamentous ascomycete fungi.

**Fig 11:**
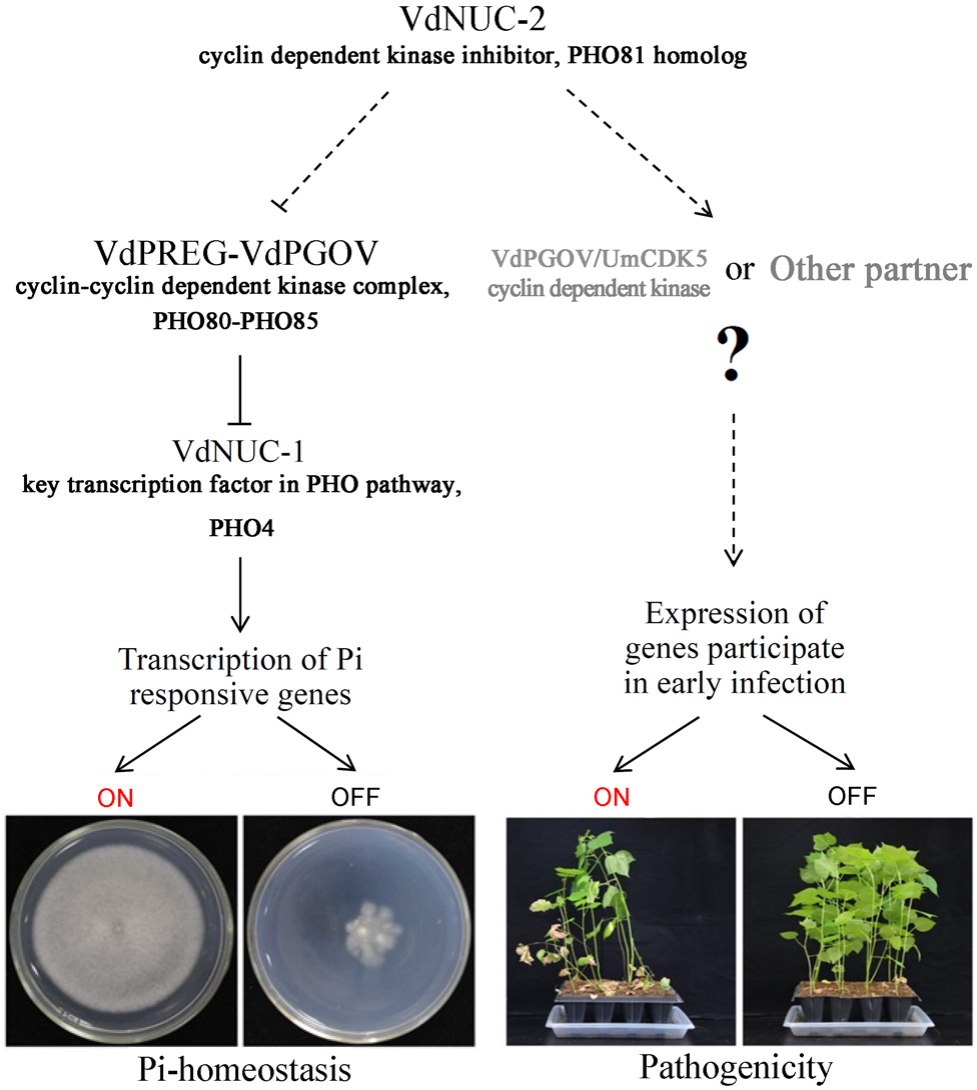
Putative schematic of the signaling pathways controlled by VdNUC-2 in *V*. *dahliae*. In *V*. *dahliae*, VdNUC-2, VdPREG, VdPGOV, and VdNUC-1 correspond to homologs PHO81, PHO80, PHO85, and PHO4, respectively, in *S*. *cerevisiae*. UmCDK5 is the homolog to VdPGOV and plays an important role in the pathogenicity of *Ustilago maydis*. Dotted lines indicate probable or unidentified interactions or pathways. The putative interacting partners are shown in gray.

To further clarify the reasons underlying the virulence defects in the *VdNUC-2* mutants, additional investigations were performed. The balance and concentration of cytosolic Pi are reportedly important for carbon metabolism in *S*. *cerevisiae* [29], and carbon metabolism or utilization is indispensable for the virulence of pathogens in *M*. *oryzae* and *V*. *dahliae* [22,23]. To investigate the role of *VdNUC-2* in carbon utilization during growth on Pi-limited conditions, we compared the radial growth patterns of the *VdNUC-2* mutant 6C4, deletion mutants *VdNUC-2Δ5*, ectopic transformant VdNUC-2C7, and wild-type strain V07DF2 under different carbon sources (sucrose, cellulose, and pectin) (Fig 3 and S5 Fig). All the strains exhibited similar radial growth patterns on the pectin plates (S5 Fig). By contrast, in the cellulose and sucrose plates, the growth of all the mutants was significantly reduced compared with V07DF2 and the ectopic transformant (Fig 3 and S5 Fig). The *VdNUC-2* mutants displayed different traits during growth under Pi starvation conditions, such as low conidia production and high sensitivity to hydrogen peroxide stress (Fig 4 and S4 Fig). Free and accessible inorganic phosphate is known to be limited in soil [30]. Thus, the abovementioned traits of the *VdNUC-2* mutants in soil are disadvantageous for infection. Moreover, Pi is involved in phosphorylation and dephosphorylation mediated by protein kinases and phosphatases, respectively [31]. Both of these processes are crucial for the pathogenicity of phytopathogenic fungi [17,32]. These findings seemed to imply that the virulence reduction in *VdNUC-2* mutants was due to the disturbance of Pi homeostasis. However, the facts, which are the extra Pi failed to restore the virulence of 6C4 (Fig 7) and *VdNUC-1* deletion in the wild-type strain did not significantly affect the virulence (Fig 8), indicated that an unknown pathway controlled by VdNUC-2 but independent of VdNUC-1 exists and is required for the host infection (Fig 11).

Similar to the homologs NUC-2 and PHO81, VdNUC-2 is a cyclin-dependent kinase (CDK) inhibitor. In *S*. *cerevisiae*, CDK inhibitor PHO81 interacts with PHO80–PHO85, the cyclin–CDK complex, to regulate the activities of the complex [33]. Castillo-Lluva et al. found that UmCDK5, a Cdk5/Pho85-like kinase from *Ustilago maydis*, is essential for full virulence, probably because mutant cells are unable to maintain the polar growth required for virulent structure formation [34]. Based on the data retrieved from a protein BLAST search at NCBI, we found that UmCDK5 (accession no. XP_011388110) was highly identical to PHO85 (*S*. *cerevisiae*, 61% similarity, CAA68773), PGOV (*N*. *crassa*, 66% similarity, XP_958274), and VdPGOV (*V*. *dahliae*, 66% similarity, XP_009657622). The conservation of the protein sequences implied that the four CDKs potentially served similar functions. Additionally, the VdNUC-2–eGFP fusion protein was found to localize in both the cytoplasm and nucleus (Fig 10B), which was completely consistent with the UmCDK5–GFP subcellular distribution as described previously [34]. In *V*. *dahliae*, the results obtained from the virulence assessments suggested that some important armaments or structures required for penetrating the host root surface were impaired because of *VdNUC-2* mutation (Fig 6). Considering the above evidence, we hypothesized that VdPGOV played a role similar to UmCDK5 in fungal virulence, but cooperation with the CDK inhibitor VdNUC-2 was critical for its normal function (Fig 11).

Ankyrin repeat motifs are found in the NUC-2 orthologs and other CDK inhibitors (Fig 2A and S2 Fig) [35]. Through the motif, CDK inhibitors interact with downstream CDKs to regulate their activity [33,35]. The members of the ankyrin repeat proteins are involved in many cellular processes, such as cell cycle, cell development and differentiation, transcriptional regulation, plant immunity, bacterial infection, and nutrient transportation [15,36–40]. The ankyrin repeat domain often co-exists with other protein functional domains, such as the PEST, calmodulin-binding, SPX, and ring finger domains [26,41,42]. An ankyrin protein may also bind to multiple targets to manipulate or cross-link distinct pathways [36]. For example, CDK inhibitor p16, a member of INK4 proteins, interacts with four partners, namely, CDK4, CDK6, NFκB, and c-Jun kinase [43]. CDK4, CDK6, and NFκB play important roles in the cell cycle [43,44], whereas c-Jun kinase participates in stress response and cell transformation [45,46]. In *Arabidopsis thaliana*, ankyrin repeat protein BDA1, which is essential for the plant’s immunity, is likely involved in two independent defense pathways (basal defense pathway and NPR1-dependent salicylic acid signaling pathway) by interacting with potentially different downstream proteins [47].

Recently, in *N*. *crassa*, Gras et al. confirmed that MAK-2, downstream of NUC-2, is involved in the hierarchical activation of the PHO pathway by negatively regulating the activity of the PREG–PGOV complex [14]. However, the molecular mechanisms under such regulation have not been fully elucidated [14]. Moreover, the transcript accumulations of the three protein kinase genes *mak-2*, *nrc-1*, and *mek-2* are regulated by NUC-2 [14,48]. In *V*. *dahliae*, *VdNUC-2* deletion would cause a virulence defect if the three homologous protein kinases are present and involved in the pathogenicity processes.

During the early stage of infection, phytopathogenic fungi use their diversified weapons to overcome the physical and immune barriers of host plants. These weapons include cell wall-degrading enzymes [1–3], special cell structures for penetration [49], host tissue-binding proteins [8], ROS-degrading system [50], and various effectors [49]. Successful attachment to host surface and penetration are key steps for the colonization and pathogenic induction of phytopathogens [12]. Some of the key equipment required for penetrating host physical barriers were likely impaired in the *VdNUC-2* mutants. Additional research is necessary to fully clarify this phenomenon. VdNUC-2 and the PHO pathway are essential for the formation of microsclerotia under Pi-limited conditions (Fig 9). Thus, some key genes in the PHO pathway might serve as potential targets to the new fungicides for *Verticillium* wilt control.

## Materials and Methods

All studies included in the present article were performed in the Institute of Plant Protection, Jiangsu Academy of Agricultural Sciences (longitude and latitude: 32.037217, 118.866713).

### Strains, media, and growth conditions

The wild-type *V*. *dahliae* strains V07DF2 and Bp2 were both isolated from infected cotton in Jiangsu Province, China. (For scientific research, the cotton field is open and can be entered without any permission. Our studies did not involve endangered or protected species.) V07DF2 did not form any visible black microsclerotia or similar resting structures in the artificial culture media [25], but Bp2 exhibited normal microsclerotial development in culture media. Potato dextrose broth/agar (PDB/PDA), Czapek–Dox media, low Pi Czapek–Dox media, and Pi-free Czapek–Dox media were employed in the study. Regular Czapek–Dox media contained sodium nitrate (23.5 mM), dipotassium hydrogen phosphate (Pi, 5.7 mM), potassium chloride (6.7 mM), magnesium sulfate (4.16 mM), ferrous sulfate (65.8 μM), sucrose (30 g/L), and agarose powder (for solid media, 10 g/L). Low Pi and Pi-free Czapek–Dox media possessed the same compositions except for the presence of 57 μM and 0 mM dipotassium hydrogen phosphate, respectively. All strains were grown in liquid medium at 25°C at 150 rpm or on plates at 25°C.

### Assessment of radial growth and conidia production

Radial growth was monitored by plate punctures at the center of the Czapek–Dox agar plates (including the low Pi and Pi-free plates). Colony diameters were measured every 3 days. All strains were incubated on plates at 25°C. To estimate conidia production, 100 μl of 1 × 10^7^ spores/ml fungal suspension was added to 50 ml of liquid Czapek–Dox medium (or low Pi Czapek–Dox medium) and then maintained at 25°C at 150 rpm for two weeks. The concentration of conidia in each liquid culture was measured by a hemocytometer.

### T-DNA insertion, targeted gene deletion, and complementation

The mutant 6C4 was obtained from our previous T-DNA insertion mutagenesis experiments [25]. In the mutant, the exon of *VdNUC-2* (VdLs.17 homolog VDAG_00896) was truncated by T-DNA (S1 Fig). The deletion construct for *VdNUC-2* knockout was prepared in accordance with the protocol reported by Paz et al. [51] and then used in the ATMT of V07DF2 to obtain independent mutant strains. A 7.0 kb genomic DNA fragment, which included the putative promoter and coding sequence of *VdNUC-2*, was amplified and cloned into the complementation plasmid named pCambia1300-ble (S6 Fig) by ClonExpress^TM^ II One Step Cloning Kit (Vazyme, Nanjing, China). The plasmid was used in the ATMT of 6C4 to generate *VdNUC-2* complementation. All primers used for plasmid construction are shown in S1 Table. Additionally, the *VdNUC-1* and *VdPHO-2* (VdLs.17 homolog VDAG_03154 and VDAG_01304) targeted deletion mutants were also obtained using the same protocol with the specific primers shown in S1 Table.

### Plant infection assay

Cotton (upland cotton cultivar Simian 3) and tobacco seedlings (*N*. *benthamiana*) were grown on a mixture of sterile roseite and nutrient soil (5:1) in a growth chamber at 25°C under a 12 h (light)/12 h (dark) photoperiod. When the second true leaves had expanded, the cotton and tobacco seedlings were inoculated with the conidia suspension (1 × 10^7^ conidia/ml) obtained from one-week-old PDB cultures. In the virulence assay of each strain, 12 to 16 seedlings were inoculated with the condia suspension by root irrigation around the stem base (25 ml per plant). For root dipping inoculation, plants were uprooted carefully, and the roots were rinsed in water. Subsequently, the roots were dipped in the conidia suspension (1 × 10^7^ conidia/ml) for 3 min. Afterward, the seedlings were replanted in soil. The disease symptoms were examined at 7, 14, 21, 28, 35, and 42 dpi, and the disease severity index was counted in accordance with our previously reported method [25]. Seven weeks later, the disease symptoms were photographed. All the inoculation experiments were repeated three times independently. Additionally, isolation of the fungi from the infected plants was performed as previously conducted by Deng et al. [25].

For the soilless plant cultures, Hoagland hydroponic nutrient solution was applied [52] and refreshed every 4 days. When the second true leaves had expanded, the cotton seedlings were transferred into the nutrient solution (with 1 mM or 6 mM Pi) containing 1 × 10^7^ conidia/ml for inoculation. After 4 days, the inoculation solution was replaced with corresponding normal Pi (1 mM Pi) or high Pi (6 mM Pi) Hoagland nutrient solution. The disease symptoms were examined seven weeks later, and the disease severity index was counted as described by Deng et al. [25].

### Genomic DNA extraction and Southern blot analysis

Genomic DNA isolation and Southern blot analysis were performed in accordance with a previous method [25]. Genomic DNA (approximately 10 μg) extracted from 6C4 was digested with *Bam*HI and *Hin*dIII, and DNA isolated from *VdNUC-2Δ5* and *VdNUC-2Δ14* was digested with *Hin*dIII and *Eco*RI. The restriction enzymes were purchased from New England Biolabs, Inc.

### RNA extraction, reverse transcription, quantitative real-time PCR (qPCR), and semi-quantitative PCR

Total RNA was isolated using TRIzol reagent (Invitrogen, Carlsbad, CA, USA) in accordance with the manufacturer’s instructions. A piece of cellophane membrane (approximately 9 cm in diameter) was placed directly on the surface of each solid culture plate (normal or low Pi Czapek–Dox plate), and the spores of each fungal strain were spread on the cellophane. One week later, the hyphae of each strain were collected from the cellophane membrane surface for RNA isolation. The details of reverse transcription and qPCR were executed as in our previous report [25]. The qPCR reaction was performed three times for each gene in each biological repeat. For data analysis, mean threshold cycle (Ct) values were calculated for each gene. The results from three independent biological repeats were similar, and one of these results was shown in histograms as representative. qPCR was performed in a Bio-Rad iQ5 thermal cycler. The results were analyzed using iQ5 software (Bio-Rad). Ex Taq DNA polymerase (TaKaRa, RR001A) was employed in semi-quantitative PCR for targeted gene detections. The primers utilized for qPCR and semi-quantitative PCR are shown in S1 Table.

### Alignment of the deduced amino acid sequences and phylogenetic analysis of *NUC-2* genes

The deduced amino acid sequences of orthologs of VdNUC-2 were downloaded from the NCBI website and aligned by MUSCLE (http://www.ebi.ac.uk/Tools/msa/muscle/) with ClustalW output format. The output data were presented using ClustalX 1.8.2. Phylogenetic trees were drawn using MEGA 5.0 software [53] on the basis of the neighbor-joining method [54]. Phylogeny was tested by the bootstrap method with 1000 replicates. The p-distance was set for the substitution model of amino acids, and gaps or missing data were treated with pairwise deletion [9].

### Full-length cDNA cloning of *VdNUC-2*

The full-length cDNA of *VdNUC-2* was obtained using the SMARTer™ RACE cDNA Amplification Kit (Clontech, 634923) in accordance with the manufacturer’s instructions. The resulting nucleotide sequence was submitted to the GenBank database (accession no. KT454782). The primers involved in *VdNUC-2* cloning are shown in S1 Table.

### DAPI staining and subcellular localization of VdNUC-2

The mycelia and conidia obtained from the surface of culture plates were stained with 2.5 μg/ml DAPI solution (in PBS) for 20 min. The samples were then rinsed twice in PBS solution before observation. DAPI was obtained from Sigma. The cellular localization of VdNUC-2-eGFP was examined by confocal fluorescent microscopy (Carl Zeiss LSM710, Germany).

## Supporting Information

S1 Fig The flank sequence of T-DNA insertion site in mutant 6C4. The flank sequence of T-DNA insertion site in mutant 6C4 obtained by a thermal asymmetric interlaced PCR. The capital letters indicated the left border sequence of the T-DNA, and the lower-case letters meant the genomic DNA of *Verticillium dahliae* strain V07DF2.
(TIF)

S2 Fig Alignments of the deduced amino-acid sequences of VdNUC-2 in comparison to homologs from other fungi species. (TIF)

S3 Fig The expression levels of *VdNUC-2* and of some other downstream genes in the tested strains growth on Czapek-Dox or on low-Pi Czapek-Dox plates. Quantitative real-time PCR was used to measure gene expression levels in wild-type strain (V07), T-DNA insertion mutant (6C4), a targeted deletion mutants (*VdNUC-2Δ5*) and two ectopic transformants (VdNUC-2C6 and VdNUC-2C7). Letter C indicated the normal Czapek-Dox media, and low-Pi meant the Czapek-Dox media with low level of Pi. *VdNUC-2* (VdLs.17 homolog VDAG_00896), *VdNUC-1* (VdLs.17 homolog VDAG_03154), *VdPHO-2* (VdLs.17 homolog VDAG_01304), *VdPHO-3* (VdLs.17 homolog VDAG_06414) and *VdPREG* (VdLs.17 homolog VDAG_06766).
(TIF)

S4 Fig *VdNUC-2* is required for H_2_O_2_ detoxification when *Verticillium dahliae* grown in Pi-deficient conditions. V07DF2: Wild-type strain; 6C4: *VdNUC-2* T-DNA insertion mutant; *VdNUC-2Δ5*, *VdNUC-2Δ14*: *VdNUC-2* targeted deletion mutants; VdNUC-2C6, VdNUC-2C7: ectopic complementation transformants. The same spore numbers (1×10^6^ spores) of each strain were spread on Czapek-Dox plates (Con _Pi_ = 5.7 mM) and on low Pi Czapek-Dox plates (Con _Pi_ = 57 μM). After that, sterile filter paper discs of 8 mm diameter were placed in the center of the plates and 15 μl of 5% H_2_O_2_ were added on them. The plates were incubated at 25°C for 6 d and the inhibition zones were measured. The error bars were calculated from the data for at least three replicates.
(TIF)

S5 Fig Growth of *Verticillium dahliae* wild-type strain (V07DF2), *VdNUC-2* mutants (6C4 and *VdNUC-2Δ5*), and complementation strains (VdNUC-2C7) on two other carbon source plates. The sucrose in low Pi Czapek-Dox medium was replaced by cellulose or pectin (10 g/L). The pH of the two medium was adjuscted to 7.0. The pictures and the diameters of colonies were obtained after two weeks of incubation. The error bars indicate standard deviations calculated from 3 replicates.
(TIF)

S6 Fig The construction of pCambia1300-ble. The gene complementation vector, designated pCambia1300-ble, was derived from the backbone of pCAMBIA1300 (CAMBIA, Canberra, Australia) (Mullins et al. 2001), in which the original hygromycin B resistance cassette was replaced by bleomycin resistance gene downstream of the trpC promoter.
(TIF)

S7 Fig Disease symptoms of soilless-culture cotton seedlings seven weeks after inoculation. Normal-Pi indicated the Hoagland hydroponic nutrient solution with ordinary phosphate level (1 mM), and high-Pi indicated 6 mM of phosphate presented in nutrient solution. V07DF2, wild-type strain; 6C4, *VdNUC-2* T-DNA insertion mutant; Control, water treatment.
(TIF)

S1 Table Primers used in the present study. (DOC)
