# Supplementary figures and images for: VdNUC-2, the Key Regulator of Phosphate Responsive Signaling Pathway, Is Required for Verticillium dahliae Infection
Source: PLoS One. 2015 Dec 15;10(12):e0145190. doi: 10.1371/journal.pone.0145190 (PMC4682923; doi:10.1371/journal.pone.0145190)

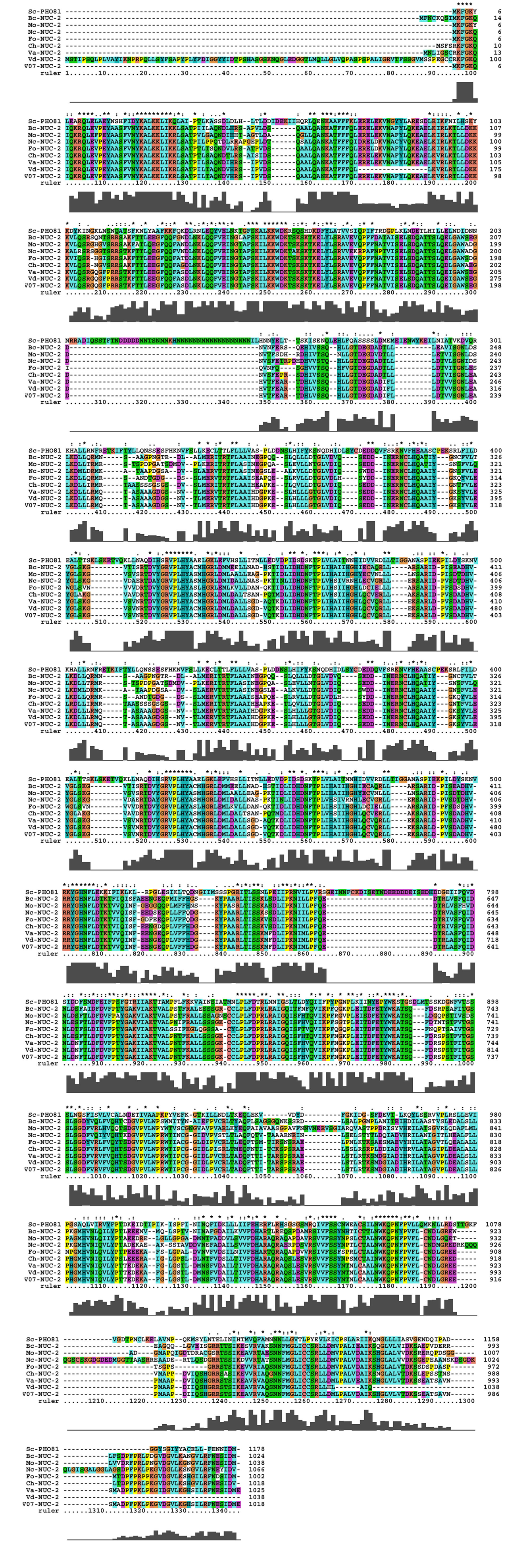

Supplement: S2 Fig — (TIF) [file pone.0145190.s002.tif]

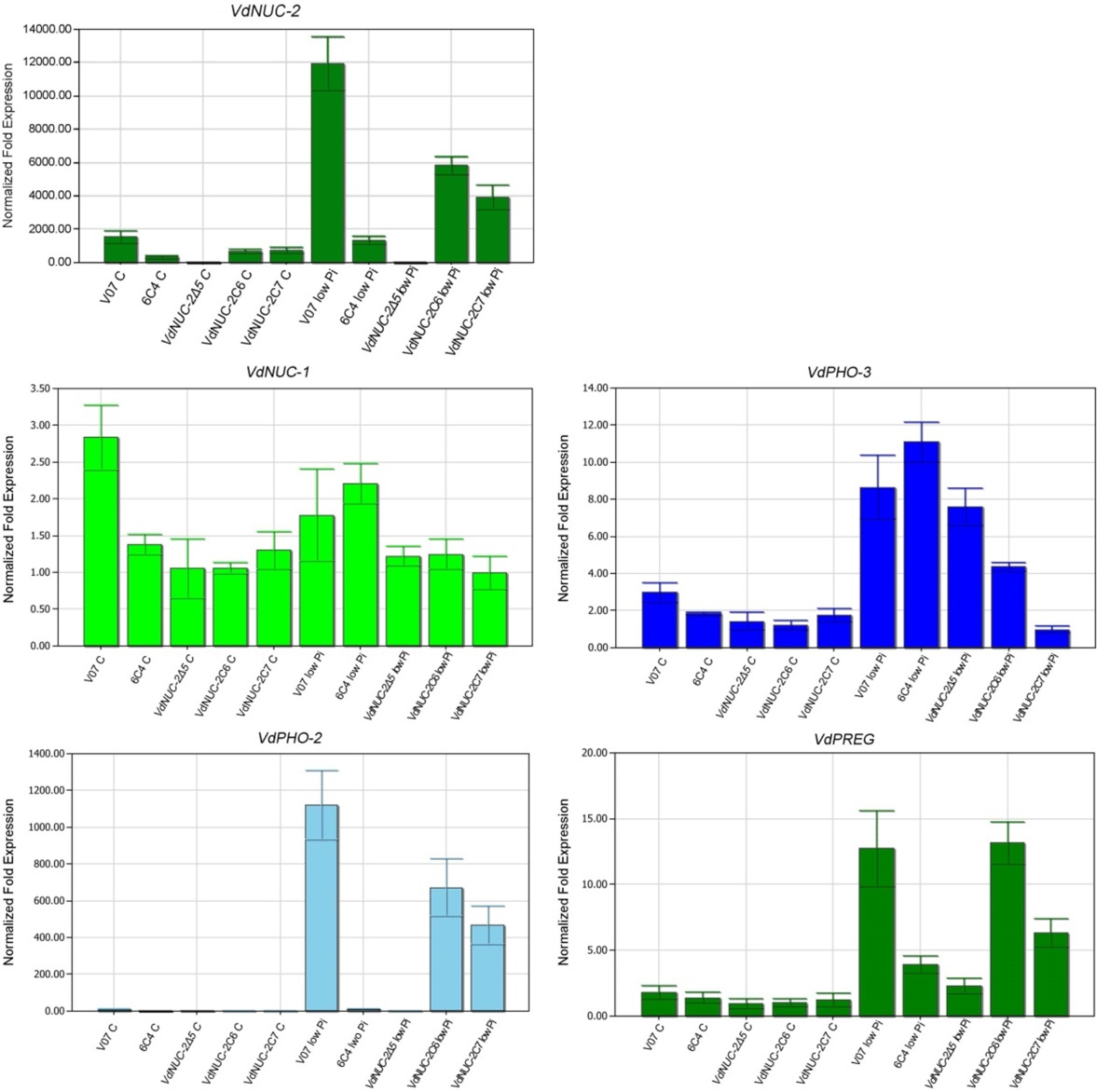

Supplement: S3 Fig — Quantitative real-time PCR was used to measure gene expression levels in wild-type strain (V07), T-DNA insertion mutant (6C4), a targeted deletion mutants (VdNUC-2Δ5) and two ectopic transformants (VdNUC-2C6 and VdNUC-2C7). Letter C indicated the normal Czapek-Dox media, and low-Pi meant the Czapek-Dox media with low level of Pi. VdNUC-2 (VdLs.17 homolog VDAG_00896), VdNUC-1 (VdLs.17 homolog VDAG_03154), VdPHO-2 (VdLs.17 homolog VDAG_01304), VdPHO-3 (VdLs.17 homolog VDAG_06414) and VdPREG (VdLs.17 homolog VDAG_06766). (TIF) [file pone.0145190.s003.tif]

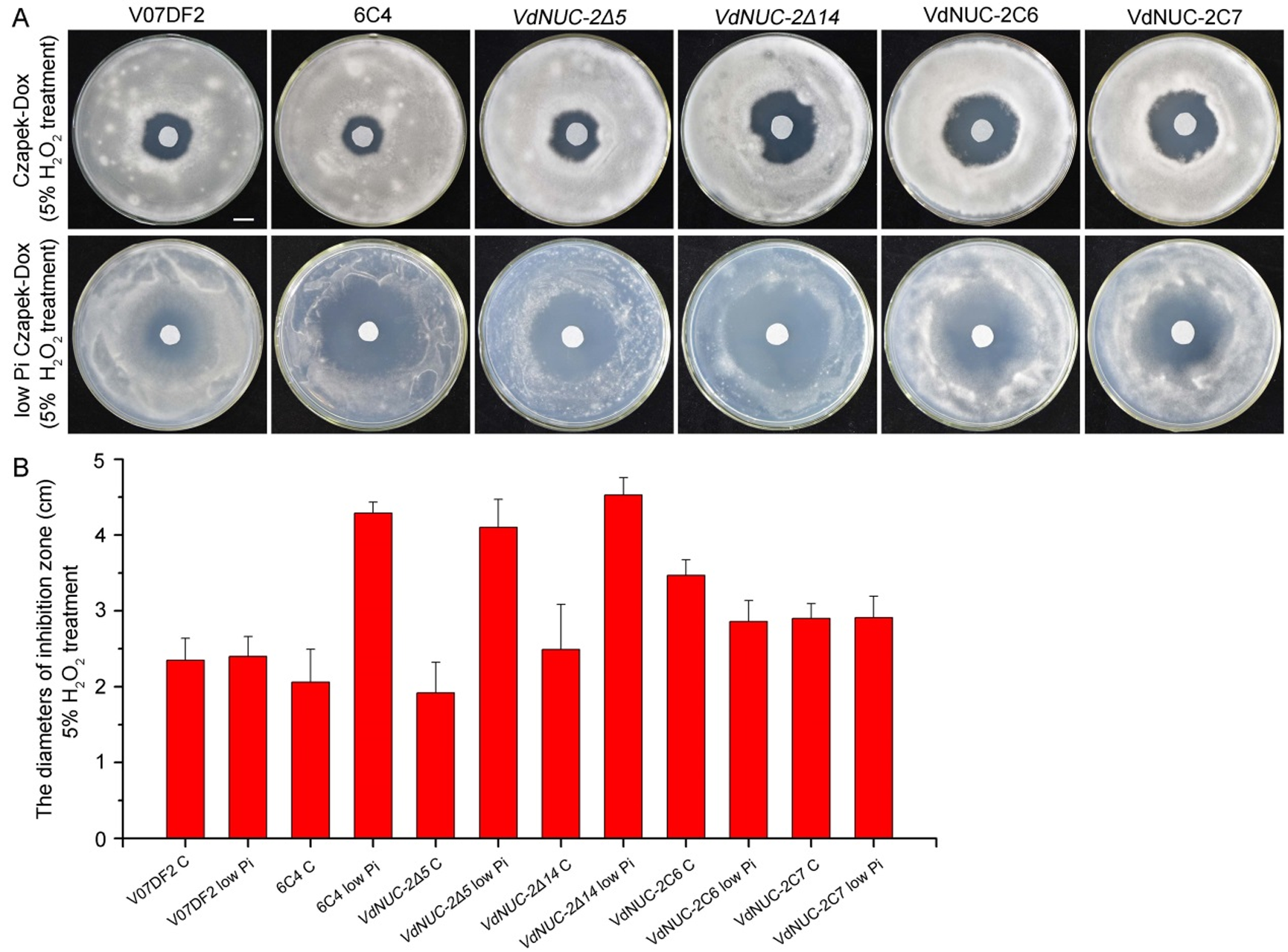

Supplement: S4 Fig — V07DF2: Wild-type strain; 6C4: VdNUC-2 T-DNA insertion mutant; VdNUC-2Δ5, VdNUC-2Δ14: VdNUC-2 targeted deletion mutants; VdNUC-2C6, VdNUC-2C7: ectopic complementation transformants. The same spore numbers (1×10^6^ spores) of each strain were spread on Czapek-Dox plates (Con Pi = 5.7 mM) and on low Pi Czapek-Dox plates (Con Pi = 57 μM). After that, sterile filter paper discs of 8 mm diameter were placed in the center of the plates and 15 μl of 5% H2O2 were added on them. The plates were incubated at 25°C for 6 d and the inhibition zones were measured. The error bars were calculated from the data for at least three replicates. (TIF) [file pone.0145190.s004.tif]

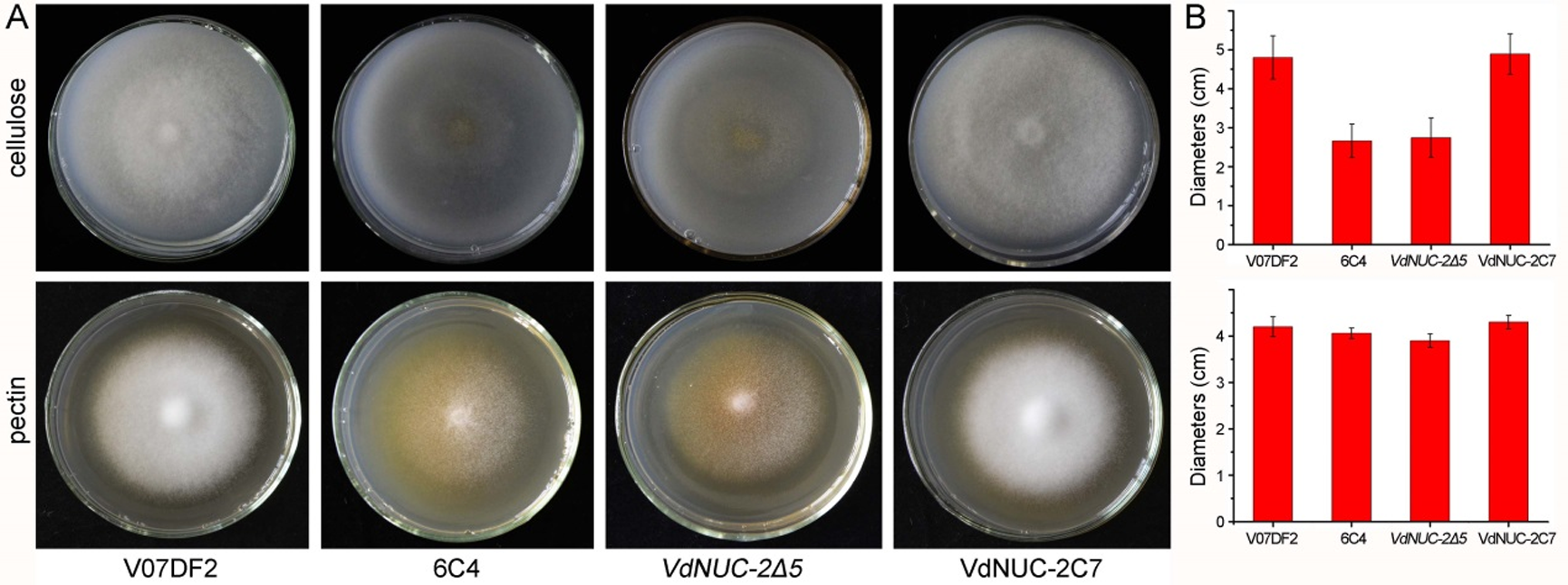

Supplement: S5 Fig — The sucrose in low Pi Czapek-Dox medium was replaced by cellulose or pectin (10 g/L). The pH of the two medium was adjuscted to 7.0. The pictures and the diameters of colonies were obtained after two weeks of incubation. The error bars indicate standard deviations calculated from 3 replicates. (TIF) [file pone.0145190.s005.tif]

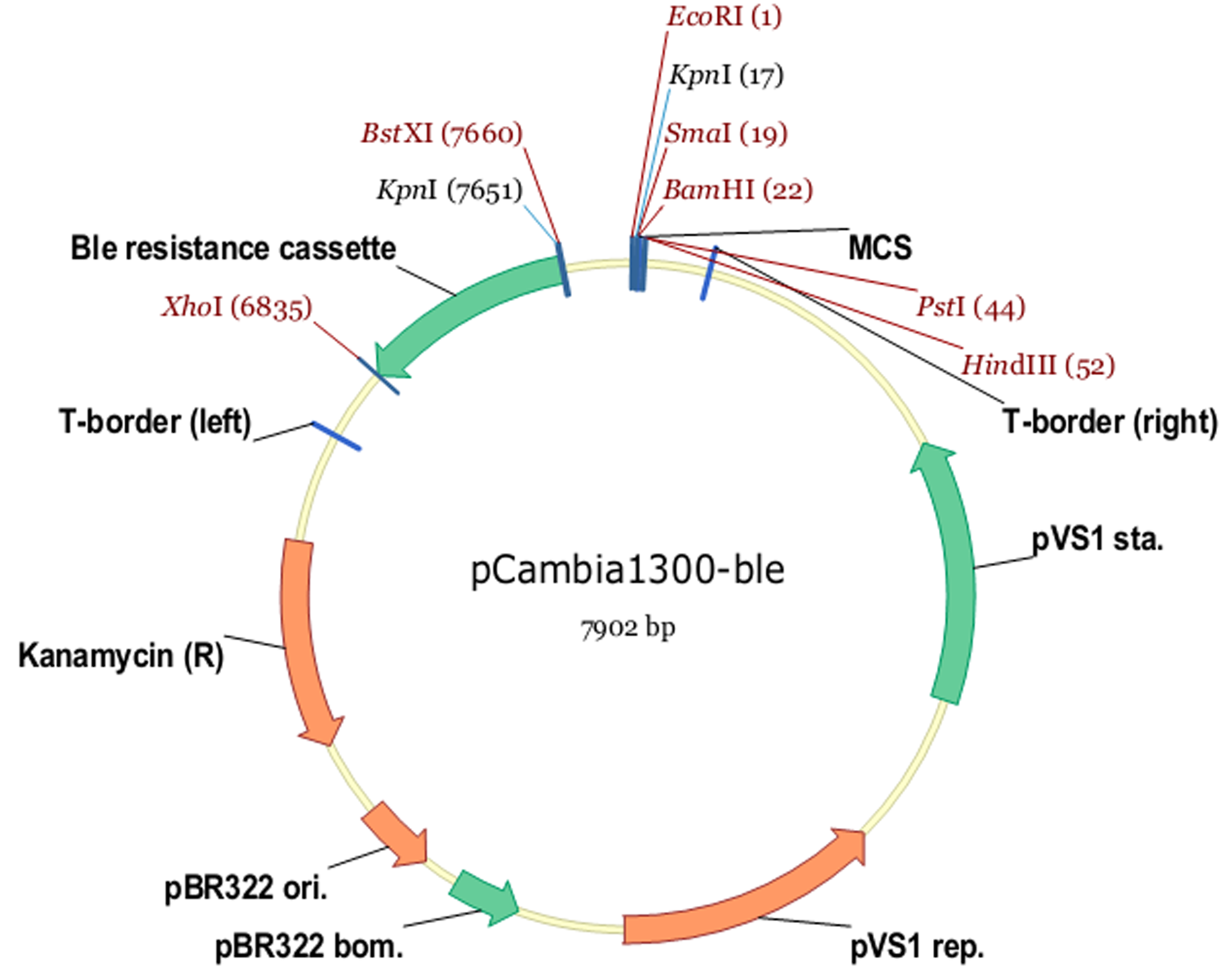

Supplement: S6 Fig — The gene complementation vector, designated pCambia1300-ble, was derived from the backbone of pCAMBIA1300 (CAMBIA, Canberra, Australia) (Mullins et al. 2001), in which the original hygromycin B resistance cassette was replaced by bleomycin resistance gene downstream of the trpC promoter. (TIF) [file pone.0145190.s006.tif]

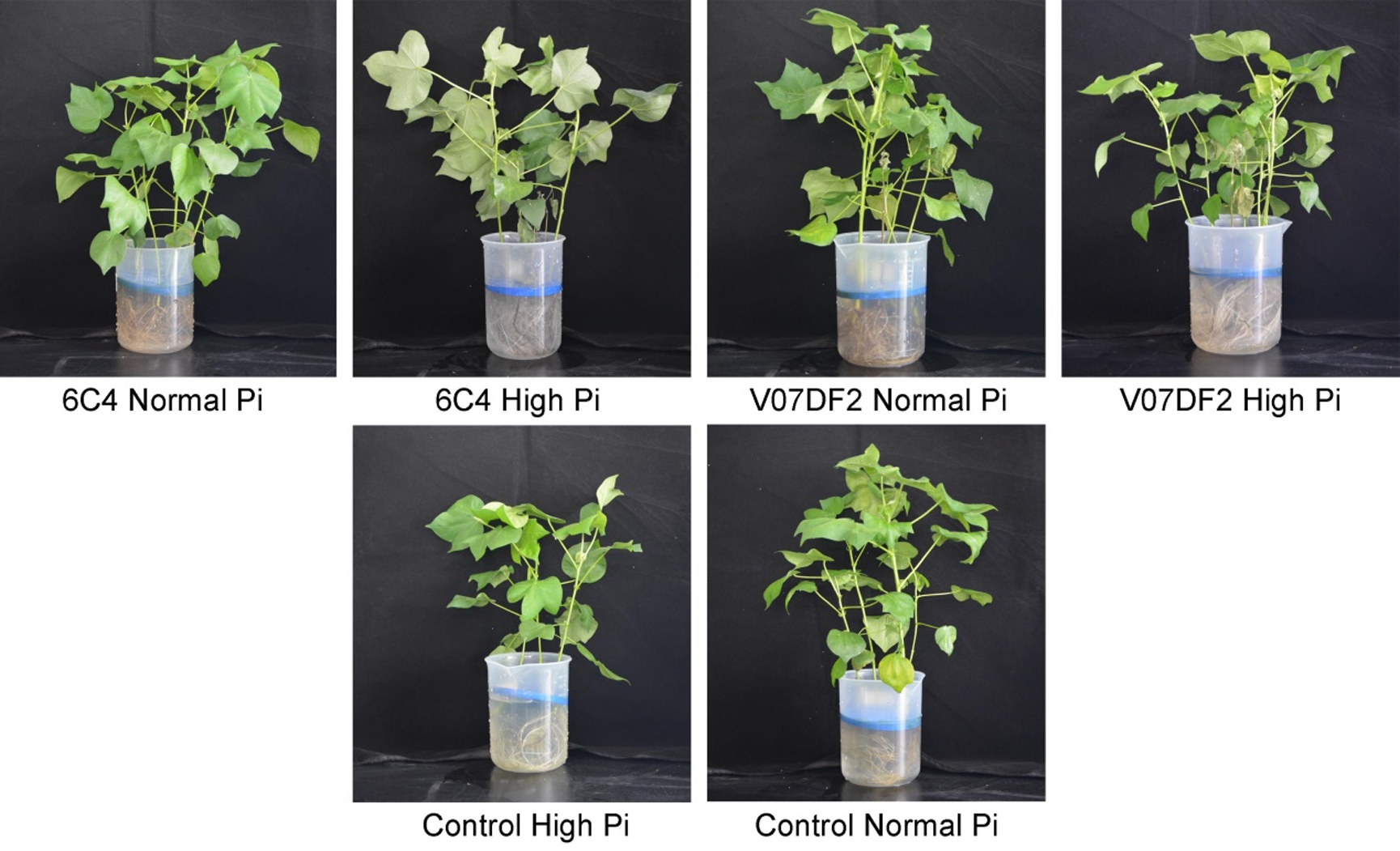

Supplement: S7 Fig — Normal-Pi indicated the Hoagland hydroponic nutrient solution with ordinary phosphate level (1 mM), and high-Pi indicated 6 mM of phosphate presented in nutrient solution. V07DF2, wild-type strain; 6C4, VdNUC-2 T-DNA insertion mutant; Control, water treatment. (TIF) [file pone.0145190.s007.tif]
