# Supplementary material for: VdNUC-2, the Key Regulator of Phosphate Responsive Signaling Pathway, Is Required for Verticillium dahliae Infection
Source: PLoS One. 2015 Dec 15;10(12):e0145190. doi: 10.1371/journal.pone.0145190 (PMC4682923; doi:10.1371/journal.pone.0145190)
Supplement: S1 Table — (DOC) [file pone.0145190.s008.doc]

| **S1 Table. Primers used in the present study** | |
| --- | --- |
| Sequences (5′→3′) | Functions |
| CCATGATTACGAATTCAGGACAAGTCAGGCTCGAAGGA  TACCGAGCTCGAATTCGCGAAACCACAGAACAAGAATC | Construction of *VdNUC-2* complementation plasmid |
| | GGGGACAGCTTTCTTGTACAAAGTGGAAGC | | --- | | GGGGACTGCTTTTTTGTACAAACTTGTCGTAGCACGTGGGTGTGATCCTAGCAGGGTTTCTCAC | | *VdNUC-2* 5′ flank sequence for targeted deletion |
| | GGGGACAACTTTGTATAGAAAAGTTGTTCCAAGTCGATGGACGGTATC  GGGGACAACTTTGTATAATAAAGTTGTTCGTCTACACTGTTGTTCTTG | | --- | | *VdNUC-2* 3′ flank sequence for targeted deletion |
| | GGGGACAGCTTTCTTGTACAAAGTGGAATCTTCCCAGTCGCTCGCTCT  GGGGACTGCTTTTTTGTACAAACTTGTCTGTGGCATCATGGAGGAGT | | --- | | *VdNUC-1* 5′ flank sequence for targeted deletion |
| | GGGGACAACTTTGTATAGAAAAGTTGTTATGCCCAGAGTGAGTCCAAC  GGGGACAACTTTGTATAATAAAGTTGTGAGACTTGGCTAGAGCTGAT | | --- | | *VdNUC-1* 3′ flank sequence for targeted deletion |
| | GGGGACAGCTTTCTTGTACAAAGTGGAAACTGGCAGGGTTCGATAGCT  GGGGACTGCTTTTTTGTACAAACTTGTCATGCCGTCACCTAGAAAAG | | --- | | *VdPHO-2* 5′ flank sequence for targeted deletion |
| | GGGGACAACTTTGTATAGAAAAGTTGTTACCGCAACGTCTACACCAAC  GGGGACA  CTTTGTATAATAAAGTTGTCTTACAAGGGCTGTTGCTAT | | --- | | *VdPHO-2* 3′ flank sequence for targeted deletion |
| | TTCCAGGGCCAGTTCATCTCG  GCGGCCGTAAAGCTTGTTGT | | --- | | qPCR for V07-*VDAG_03222* |
| | AAGAGCTGGCCTACATGCGT  TATGTGGCGCTGAGAGCAGT | | --- | | qPCR for V07-*VDAG_03800* |
| | ACCGTCTGTACGCTGTCGAT  AGAG | | --- |   CAGAGCGAAACCAGT | qPCR for V07-*VDAG_07583* |
| | CACCGCTCAGAATGACGTTCAC  TCTTGTCGAGGAGCGTTCTCAG | | --- | | qPCR for *VdNUC-2* |
| | TGTGCGCCAGTCTCCAATCT  TTCGGGTGAGACGGAAGCAT | | --- | | qPCR for *VdNUC-1* |
| | TGCCAGCGAGGATGGTTTCT  AATCGTGGCGCGGACAGTAT | | --- | | qPCR for *VdPHO-2* |
| | ATTCCGCTCCTCAGCACGAT  TGACCTTCCAAGTGGCGATGT | | --- | | qPCR for *VdPHO-3* |
| | TCTGATCGCCCACATGCTCT  GGCGTCAATGTCGCATGCTT | | --- | | qPCR for *VdPREG* |
| | GCCCAGCAGATGTTTGACCC  TCGGTGAACTCCATCTCGTCCA | | --- | | semi-quantitative PCR for internal reference gene *β-tubulin* |
| | CCGCTCAGAATGACGTTCACC  TCGCCCTCAGACCAAGCTC | | --- | | semi-quantitative PCR for *VdNUC-2* 5′ flank sequence |
| | CGACCCTGATGCGATACCAG  CTCGCACAAAGTCGCCAGA | | --- | | semi-quantitative PCR for *VdNUC-2* 3′ flank sequence |
| GCCGTGTCTGCAGGCGCTTCTTGACG | RACE for 3′ sequence amplification of *VdNUC-2* |
| AGCAAGAGTGGGCTCTCCCGCGTCTG | RACE for 5′ sequence amplification of *VdNUC-2* |
| GACCAGCTAGACACCCAAATG  CAAGGGGGGTAAAGGCCATCT | semi-quantitative PCR for *VdNUC-1* |
| GACAGCGTCTCCGACCTGATGC  TGGGGCGTCGGTTTCCACTATC | hygromycin B resistance gene detection |
